# The Bias‐and‐Expertise Model: A Bayesian Network Model of Political Source Characteristics

**DOI:** 10.1111/cogs.70141

**Published:** 2025-11-19

**Authors:** David J. Young, Lee H. de‐Wit

**Affiliations:** ^1^ Department of Psychology University of Cambridge

**Keywords:** Bayesian, Network, Political, Source, Characteristics, Bias, Expertise, Polarization

## Abstract

Perceptions of source credibility may play a role in major societal challenges like political polarization and the spread of misinformation as citizens disagree over which sources of political information are credible and sometimes trust untrustworthy sources. Cognitive scientists have developed Bayesian Network models of how people integrate perceptions of source credibility when learning from information provided by sources, but these models do not involve the crucial source characteristic in politics: bias. Biased sources make claims that align with a particular political agenda, whether or not they are true. We present a novel Bayesian Network model which integrates perceptions of a source's bias as well as their expertise. We demonstrate the model's validity for predicting how people will update beliefs and perceptions of bias and expertise in response to testimony across two studies, the second being a preregistered conceptual replication and extension of the first.

## 1 Introduction

Psychologists have long known that who delivers a message affects whether people believe it (Harris, Hahn, Madsen, & Hsu, 2016; Hovland & Weiss, 1951; Lupia & McCubbins, 1998; Marks, Copland, Loh, Sunstein, & Sharot, 2019; Mercier, 2020). In recent years, understanding source effects has become increasingly important in attempts to understand polarization and misinformation (e.g., Young, Madsen, & de‐Wit, 2025). For example, a meta‐analysis shows that political partisans perceive claims made about politics to be more credible when they come from in‐group sources (Ditto et al., 2019). Fake news is also perceived as more credible when it comes from a mainstream news outlet (Dias, Pennycook, & Rand, 2020), or an in‐group source (Traberg & van der Linden, 2022).

While source effects are often attributed to motivated reasoning (Ditto et al., 2019) or the use of heuristics (e.g., Chaiken & Maheswaran, 1994), there is a richly developed field spanning experimental psychology and social epistemology that views a source's testimony as probabilistic evidence which it is rational to utilize if proper account is made of the characteristics of the source (Bovens & Hartmann, 2003; Collins, Hahn, von Gerber, & Olsson, 2018; Harris et al., 2016; Merdes, von Sydow, & Hahn, 2021; Young et al., 2025). Of particular interest are Bayesian Network models of source effects (Bovens & Hartmann, 2003; Collins et al., 2018; Hahn, Harris, & Oaksford, 2013; Hahn, Oaksford, & Harris, 2013; Harris et al., 2016; Madsen, 2019; Merdes et al., 2021; Olsson, 2011, 2013; Pallavicini, Hallsson, & Kappel, 2021; Young et al., 2025). These models treat learning from a source's testimony as a Bayesian inference problem^1^ where listeners try to infer the probability that the hypothesis being talked about is true, taking into consideration how the capabilities and intentions of the source should affect the testimony they give. These models have empirically‐demonstrated predictive validity (Collins et al., 2018; Harris et al., 2016; Merdes et al., 2021; Young et al., 2025).

Two prominent types of Bayesian Network models of source characteristics have been developed. One assumes that people track a source's *Reliability* (Bovens & Hartmann, 2003; Collins et al., 2018; Olsson, 2011, 2013), where the more reliable a source is, the more likely it is that their claims are true. Upon witnessing a source making a correct claim, their reliability is revised upward, and for incorrect claims, their reliability is revised downward. Alternatively, the Trustworthiness‐and‐Expertise Model (Hahn et al., 2013; Harris et al., 2016; Madsen, 2019; Madsen, Bailey, & Pilditch, 2018) distinguishes between accidental and intentional sources of error: a source's *expertise* affects their beliefs, with high expertise leading to accurate beliefs and low expertise leading to random beliefs, whereas *trustworthiness* affects whether the source is honest about what they believe or lies, saying the opposite of what they believe to be true.

These models provide explanatory theories of why people are influenced by source effects in addition to several other benefits. First, because they are computationally specified, their predictions are less ambiguous than the verbal theories typical of contemporary psychology (see Guest & Martin, 2021; Muthukrishna & Henrich, 2019; Oberauer & Lewandowsky, 2019). Therefore, precise predictions of how people should behave in given experimental scenarios can be derived *a priori* using equations and simulations. This reduces the researcher's degrees of freedom when it comes to specifying which experimental results would falsify the model as an explanatory theory, making experimental results more informative and inferences more robust.

Second, these models can be integrated into agent‐based models, where they act as models of the agents’ cognition (e.g., Hahn, Merdes, & von Sydow, 2018; Madsen et al., 2018; Olsson, 2013; Pallavicini et al., 2021; Pilditch, Roozenbeek, Madsen, & van der Linden, 2022). These agent‐based models provide insights into how complex societal patterns of belief and behavior related to source effects might arise which are not always predictable from individual‐level models due to emergence. They also provide a means for testing the possible effect of interventions or changes in background conditions, which may be difficult to study empirically.

However, existing Bayesian Network models of source cognition do not capture what we argue is the central construct of source credibility in politics: *bias*. The importance of bias can be demonstrated by a thought experiment. Imagine you witness a politician repeatedly defend a series of policies passed by their party which you knew had been disastrous. According to the existing models, this should lead you to attribute either low reliability or low trustworthiness and expertise to the politician. Now, suppose you subsequently saw the politician being asked to comment on the impact of a policy their party had passed which you knew had actually been a *success*. What would you expect them to say? You would surely expect them to *praise* the policy, because it is their party's. But this prediction is at odds with what the existing models say you should expect—these models would predict that, due to the low reliability, expertise, and trustworthiness previously evinced by the politician, you should expect the politician to make an *incorrect* claim again, and say the policy has *not* been beneficial.

We can easily generate the correct prediction, however, if we assume that what we are tracking is not (just) the politician's reliability, trustworthiness, or expertise, but their bias. When a source is biased, the claims they make are influenced by the congeniality of the claim toward a particular political perspective. We can think of a biased source as having a leaning toward one side of a particular dimension of politics, such as Left versus Right, Democrat versus Republican, Leave versus Remain, or Government versus Opposition. Possible states of the world, or “hypotheses,” can have political leanings too—for instance, the hypothesis “low corporate taxes boost economic growth” is right‐leaning because if it is true, it supports a right‐wing ideology. Biased sources have a tendency toward saying that hypotheses which lean toward the same side as them are *true*, and that hypotheses which lean toward the opposing side are *false*. The more biased the source is, the more their claims are determined by this congeniality rather than the ground truth.

Biases can arise either intentionally or unintentionally (Eagly, Wood, & Chaiken, 1978). Intentionally, sources may want to proselytize for a particular perspective and be willing to lie, or speak without regard for the truth, to do so. Unintentionally, sources may have beliefs which are “sincerely” biased toward a particular perspective (Wallace, Wegener, & Petty, 2020a) due to biases in their reasoning or the information they have relied upon to form those beliefs, leading them to make biased claims though they have no conscious desire to do so.

No existing Bayesian Network model of source characteristics integrates bias. However, empirical research has already recognized that people are influenced by perceptions of source bias. People update their beliefs less when they believe the source is biased toward making the claim (Eagly et al., 1978; Wallace et al., 2020a, 2020b), and attribute more bias to sources when they make claims that appear implausible (Eagly et al., 1978; Wallace, Wegener, Quinn, & Ross, 2021). In politics, people attribute bias to others who express political views they disagree with (Cheek, Blackman, & Pronin, 2021; Kennedy & Pronin, 2008), and after learning that the journal *Nature* had endorsed Biden for President in 2020, Republicans increased their perception that *Nature* was biased (Zhang, 2023).

Politicians, media sources, citizens, commentators, businesses, experts, and institutions may be politically biased, and they may be biased toward or against particular parties, politicians, policies, ideologies, and movements. Their biases may stem from biases in their information, their reasoning, or from their conscious intentions. Accusations and examples of each type of bias are common in political discourse, and indeed academic research; Table 1 contains numerous examples of biases alleged to systematically influence what people believe and say about politics.

**Table 1:** Examples of biases and allegations of biases in people's information, reasoning, and intentions when communicating about politics

Information	Reasoning	Intentional
Academic concern over “echo chambers” and the design of social media algorithms (Van Bavel, Rathje, Harris, Robertson, & Sternisko, 2021). Academic concern over the effects of watching Fox News (Broockman & Kalla, 2022). The claim that mainstream media organizations are used by elites to control the public through propaganda (e.g., Herman & Chomsky, 1988). Trump's claim that mainstream media is biased against him (Meeks, 2020). Allegations from US conservatives that social media platforms are biased against them (e.g., “Twitter Files”, 2023).	Terms used to disparage left‐wingers’ intelligence, like “the woke mind virus,” the “loony left,” “libt*rd,” and “Trump derangement syndrome.” Academic claims that political beliefs are distorted by social identities (Kahan, 2016; Van Bavel & Pereira, 2018). The perception that our political opponents are more prone to counter‐normative reasoning (Schwalbe, Cohen, & Ross, 2020). The alt‐right claim to being “red‐pilled,” and the left‐wing claim to being “woke,” both implying that they, unlike others, see the world for how it really is.	Trump's claim of a “Witch Hunt” against him, led by “Deep State” bureaucrats, Democratic Prosecutors, the FBI, and others. Corbyn's claim that antisemitism in Labour under his leadership was “dramatically overstated for political reasons by our opponents inside and outside the party, as well as by much of the media” (Walker & Elgot, 2020). Academic concern that political media outlets are intentionally biased in order to appeal to specific target audiences (Gentzkow & Shapiro, 2006). Widespread use of “spin” by politicians to avoid admitting uncongenial truths.

There are, therefore, multiple reasons why it would be desirable to develop a Bayesian Network model of source effects that integrates source bias. This model would provide a precise, computational, and testable explanation of why people are sensitive to source characteristics in politics generally. It would similarly provide an explanation for the known empirical phenomena concerning how people account for and infer source bias. It would also advance the literature on Bayesian Network models of source effects by providing a more valid model for applications to politics. Plus, biased sources exist in many other domains studied by psychologists, like financial negotiations, legal cases, advertising, and science communication, so a model of how people can infer and account for source bias may be usefully applied to studying how people learn from source testimony in these domains too. The next section articulates the model we have developed to fill this gap.

### 1.1 The model

The Bias‐and‐Expertise model is a model of how people update beliefs in response to source testimony, which integrates perceptions of the source's bias direction, bias intensity, and their expertise. Within Marr's framework (see, e.g., Peebles & Cooper, 2015), the model describes the *computational* level of the relevant cognitive processes—representing the reasoning problem that needs to be solved—and so it may capture variance in people's inferences even if, at the *algorithmic* level, they do not employ Bayesian reasoning, which people are often thought not to do (e.g., Edwards, 1982; Mandelbaum, 2019), though perhaps unfairly (Chater et al., 2020; Gershman, 2021; Hahn et al., 2013; Hahn & Oaksford, 2007; Oaksford & Chater, 2009, 2020; Zhu, Sanborn, & Chater, 2020). While solving problems via Bayesian inference is often computationally intractable, even to the extent of approximations being intractable (Kwisthout, Wareham, & van Rooij, 2011; van Rooij, Wright, Kwisthout, & Wareham, 2018), Bayesian inference *can* be tractable using simple Bayesian Networks with few states^2^ and interactions (Kwisthout & van Rooij, 2020), just like the Bias‐and‐Expertise model. So, if the model is correct in identifying the factors which people are sensitive to when reasoning about testimony as well as the relationships between those factors, and if people integrate information about these factors and relationships more‐or‐less in a Bayesian way, then actual cognition and the model's predictions should “meet in the middle,” making it an effective predictive model of how people will react to testimony from potentially‐biased sources, as well as a reasonable descriptive model of their cognition.

When a source makes a claim, we model this as the source providing “testimony” about a “hypothesis,” where the hypothesis is the state of the world that their claim concerns, and the testimony is whether their claim implies that it is true or false. For instance, if the claim was “Brexit has boosted the British economy,” the hypothesis would be “Brexit has boosted the British economy,” and the testimony would be modeled as “True.”

**Fig. 1:**
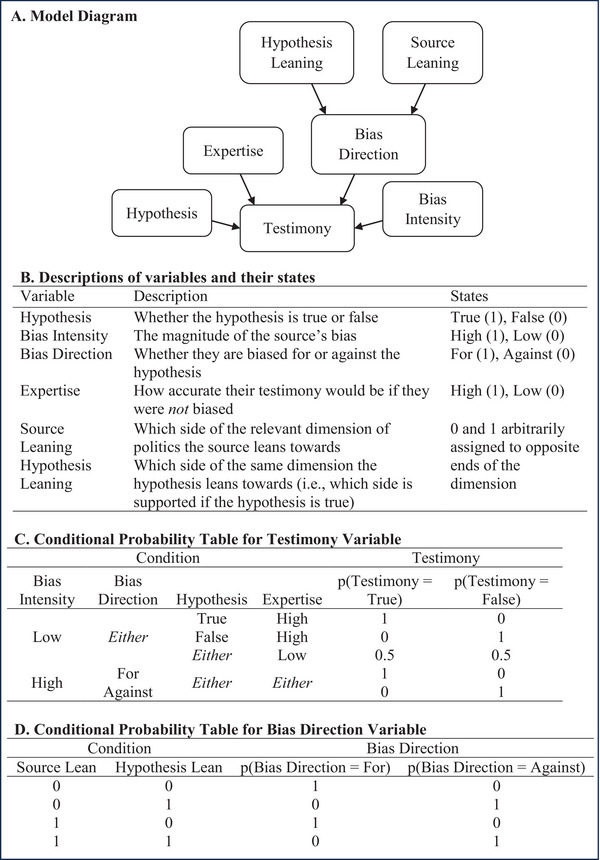
Core information about the Bayesian Network. (A) Diagram of the model in the form of a directed acyclic graph. (B) Information about each variable and its states. (C) The conditional probability table for the Testimony variable. (D) The conditional probability table for the Bias Direction variable.

As a Bayesian Network, the model specifies conditional probability relationships between variables. All variables can occupy two different states, and we model the probability of each variable occupying each state, which can be updated using Bayes’ rule. A diagram of the network is shown in Fig. 1A, and the table in panel B of Fig. 1 provides explanations of what each variable measures and its possible states. In the diagram, the arrows show which variables influence each other and in which direction—an arrow pointing from one variable to another shows that the state of the first variable, the “parent,” probabilistically influences the state of the second, the “child.” Where a variable has multiple parents, the states of the parents interact to determine the state of the child. Panels C and D of Fig. 1 show the conditional probability tables for the hypothesized relationships between variables.

As the table in Fig. 1C shows, when a source's bias intensity is 0, they are impartial, and their testimony becomes a function of only the ground truth and their expertise—experts’ testimony reflects the ground truth, whereas nonexperts testify at random. Conversely, when a source's bias intensity is 1, they are biased, and now their testimony is only a function of their bias direction—sources biased *for* the hypothesis will say it is true, and those biased *against* will say that it is false. Thus, the greater the bias intensity, the less sensitive their testimony is to the truth. As the table in Fig. 1D shows, a source's bias direction is the product of an interaction between their own leaning and the leaning of the hypothesis—when the two match, they are biased for the hypothesis, when they clash, they are biased against it.

By modeling bias intensity and bias direction separately, the model can capture the intuition that sometimes we know a source is likely to be biased without knowing which direction they are biased toward—for example, if we know we are watching an interview with a politician, but we do not know their party. Equally, we might know which direction a politician would be biased toward—toward their party, for example—without knowing just how biased they are. By also including an expertise node, we capture the intuition that impartial sources also differ in their credibility as a function of how well‐informed and intelligent they are.

To obtain predictions from the model we must input priors, which are beliefs about the states of the variables expressed as probabilities. For example, if I think the hypothesis is likely to be true but I am not totally certain, I might have a prior of 0.8, whereas if I think it is probably wrong, I might have a prior of 0.2. Whichever state of the variable corresponds to “1” in Table 1, our prior is the probability we assign to that variable occupying that state before we hear the testimony. Once priors have been set (as the prior for Bias Direction is determined by the priors for Source Leaning and Hypothesis Leaning, it does not need to be set manually), we apply Bayes’ Rule to determine how we should update them in response to hearing the source's testimony. Updating our prior beliefs gives us “posterior” beliefs, and these posterior beliefs constitute the model's predictions about the inferences that should be drawn in response to source testimony.

### 1.2 Model behavior

In Figs. 2A–E, we show how the model predicts that beliefs about each variable should be updated in response to testimony that a hypothesis is true. Code for these simulations is available in our OSF project.

Fig. 2Model Behavior when a source testifies that a hypothesis is “True,” as a function of the listener's prior belief in the hypothesis and prior perceptions of the source's characteristics. *Note*. Updating is expressed as the log (base = 2) of the likelihood ratio. (A) Belief in the hypothesis is updated in the direction of the source's testimony, and more so the higher their Expertise, but less so the higher their Bias Intensity and the more their Bias Direction is consistent with the claim. (B) Bias Intensity is updated more positively when the Hypothesis is less plausible and the source's Bias Direction is consistent with the claim; the higher the source's Expertise, the stronger the interaction with the Hypothesis prior. (C) Expertise is updated positively when the source's claim is likely to be true (i.e., Testimony = “True” and p(Hypothesis = True) > 0.5), but negatively when likely to be false (i.e., Testimony = “True” and p(Hypothesis = True) < 0.5). Both of these effects are weaker the higher the source's Bias Intensity and the more their Bias Direction is consistent with the claim. (D) Bias Direction is updated more positively when the Hypothesis is less plausible and the source's Bias Intensity is higher; the higher the source's Expertise, the stronger the interaction with Bias Intensity. (E) Sources are thought to lean in the same direction of Hypotheses when they say they are true, more so the higher their Bias Intensity and Expertise. Note that the trends are identical if the “Hypothesis Leaning” and “Source Leaning” variables are swapped over.

**Figure:**
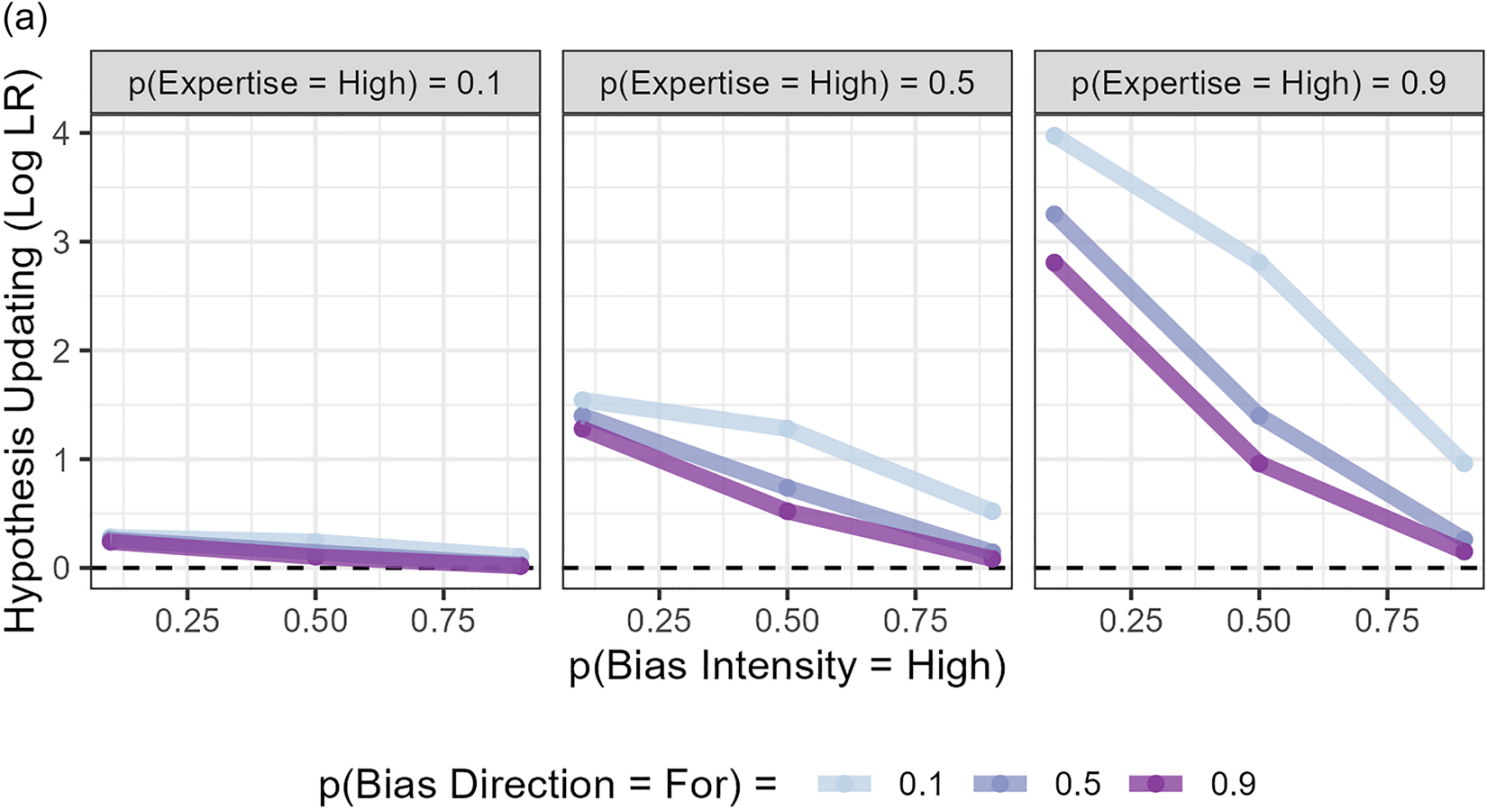

**Figure:**
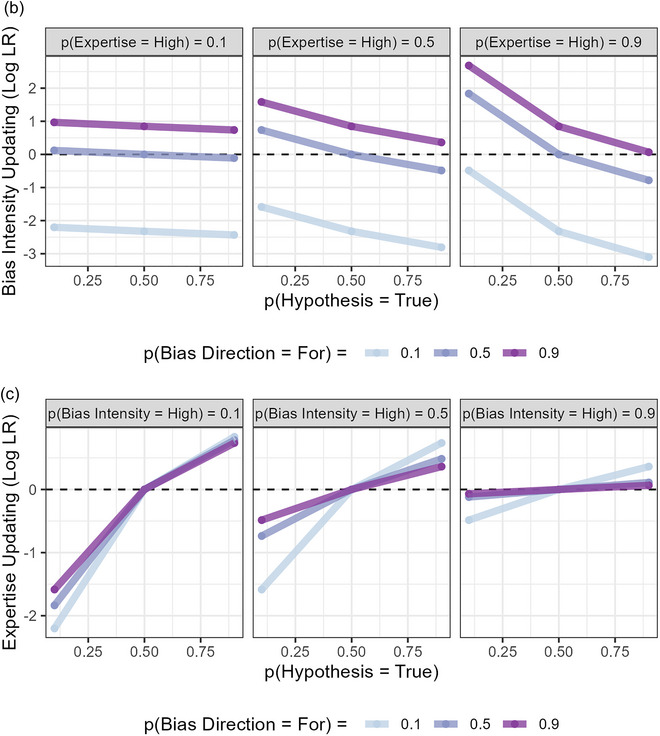

**Figure:**
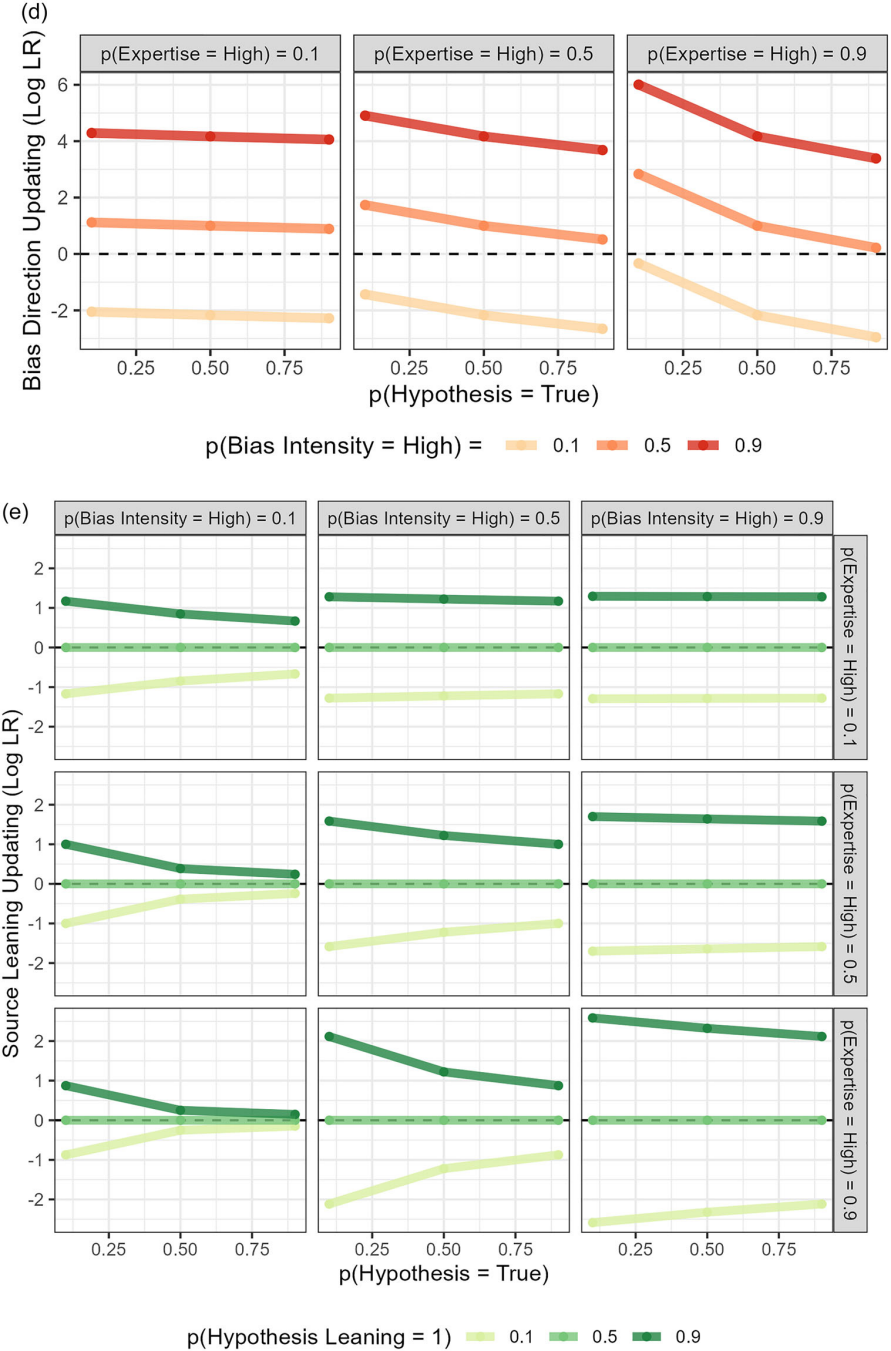

We calculate the amount of updating by finding the log likelihood ratio of the testimony. This is because in the logit^3^ form of Bayes Rule, logit(posterior) = logit(prior) + log(likelihood ratio), the log likelihood ratio is the amount we add to our (logit) prior belief in the hypothesis to obtain our (logit) posterior belief in the hypothesis after updating it to absorb the information conveyed by the evidence. It, therefore, indexes the amount of updating in an intuitive way: when it is 0, we do not update, when it is positive, we update positively, becoming more confident that what we are learning about is true, and when it is negative, we update negatively, becoming more confident that what we are learning about is false. In what follows, the likelihood ratio is how much more likely we think it is that we would observe the evidence when the variable we wish to learn about is in its “1,” “True” or “For” state rather than its “0,” “False,” or “Against” state, expressed as a ratio.

The behavior of the model shown in Fig. 2 demonstrates that it can explain several known phenomena related to source bias in the existing literature. As Fig. 2A shows, it predicts lower belief updating the more a source is thought to be biased toward the claims they make (Wallace et al., 2020a, 2020b). As Figs. 2B and C show, it predicts higher attributions of bias intensity and bias “For” the hypothesis the less we deem a claim to be plausible (Cheek et al., 2021; Kennedy & Pronin, 2008; Wallace et al., 2021; Zhang, 2023). It also offers a potential explanation for why sources are more persuasive when they adopt positions which they would normally be expected to oppose (Eagly et al., 1978; Wallace et al., 2020a; Walster, Aronson, & Abrahams, 1966)—that sources are more persuasive when the claim they make runs against the direction of their bias is predicted by the model, as Fig. 2A shows.

### 1.3 The current studies

To assess the validity of the Bias‐and‐Expertise model as a model of source effects in politics, we have conducted two experimental studies. In these studies, participants receive testimony from sources, and we manipulate factors like their prior for the hypothesis and their perceptions of the source's bias intensity, bias direction, and expertise. We measure their priors for these variables (or make reasonable assumptions about them) before revealing the testimony, and, for each individual, input them into the model to generate predictions about what their posteriors should be. We elicit posteriors directly from participants after they have received the testimony, and compare their reported posteriors to our predictions.

Like Harris et al. (2016), we use a third‐person paradigm where participants see dialogues between two characters, and it is the participant's task to estimate the priors and posteriors that *one of the characters* should hold. In our studies, the characters are discussing politics in their country, with one character telling the other what a source has said about whether a new Government policy has been damaging or beneficial for their country. The participants estimate the priors and posteriors of the character who receives the information. We always describe their country as a fictional Western democracy that is not the US or UK to avoid people's beliefs about the bias of real‐world political entities influencing their judgments.

We use fictional scenarios rather than real‐world political stimuli so that we can avoid any effects of real‐world partisanship on people's reasoning. While it will be important to assess the performance of the model in partisan contexts, it makes sense to begin by obtaining a baseline measure of the model's performance in a controlled environment. If we were to use partisan contexts from the beginning, and discovered the model performed poorly, it would be ambiguous whether this was due to the model being generally inaccurate or if its accuracy was undermined by partisanship.

By using a third‐person paradigm, we can easily manipulate priors by simply having the character makes statements which suggest particular priors (e.g. “I have no idea”), and it avoids the problem of having to ask participants for their prior opinions about completely fictional issues and sources. Moreover, participants should use their own reasoning to determine what fictional characters should believe, as long as they believe the character to be as rational as themselves.

Participants provide all their priors and posteriors using 0−100 scales. Again, this is the approach used by Harris et al. (2016). We do not believe that people's beliefs about the states of the world are stored as numerical values on a 0−100 scale, but variations in confidence should be available to introspection, and a 101‐point scale allows for high sensitivity to these variations. Responses on this scale can also be easily converted to probability estimates before entry into the model by dividing by 100. The drawback of the scale is that different people may translate the same level of introspective confidence into different points on the scale due to its abstraction. This will create a degree of between‐participant noise. However, this noise did not prevent Harris et al. (2016) from demonstrating good predictive validity for their models, so this is a limitation rather than an insurmountable problem.

We also follow Harris et al. (2016) by converting any priors of 1 to 0.99 and 0 to 0.01 before generating predictions. While in many cases, this has very little effect on the predicted posterior, the reason for doing so is that if priors of 0 and 1 are allowed, the likelihood ratio of the testimony can become infinite or undefined in some cases, leading to undefined posteriors. Probabilities of 0 and 1 imply a degree of certainty so high that it is not even theoretically possible for observational evidence to change one's mind—as Harris et al. (2016) argue, it is unlikely participants understand and intend to convey this. Converting to 0.01 and 0.99, therefore, solves the problem through minimal intervention while still representing the participant's very high degree of certainty. However, throughout these studies, we never vary the Hypothesis Leaning, as all hypotheses lean toward the Government, and so we set this prior at 1 for the sake of generating predictions. This happens not to allow for undefined posteriors to occur; we could equally have set it to 0.99, but setting to 1 was the approach we took throughout and preregistered ahead of Study 4. Changing to 0.99 has a negligible impact on the performance of the models.

We use three measures to assess the performance of our model. The first is the correlation between observed and predicted posteriors at the individual level. In order to have any claim to predictive validity, our model's predictions should correlate with individual observations. The second measure is one used by Harris et al. (2016) and Madsen (2019), which we call Variance in Means (VM). To calculate VM, we first calculate the mean predicted posterior in every cell of the study design, as well as the mean observed posterior, and then calculate the amount of variance in the observed mean posteriors explained by the predicted mean posteriors, using adjusted *R*‐squared. This tells us how well the model predicts differences in aggregate belief across conditions. For a benchmark, Harris et al. (2016) and Madsen (2019) found VM scores of 85−90%.

Our final measure is the Brier score. This is simply the mean squared difference between observed and predicted posteriors. Rather than measuring how well the model's predictions explain the *differences* between people and conditions, like correlations and VM, respectively, the Brier score is a measure of the absolute accuracy of the model—how *close* are our predictions to the observations? The Brier score has been used by Hahn et al. (2018) to assess the accuracy of Bayesian Network models in theoretical simulations. We expect high correlations and VMs, and low Brier scores. We also perform visual inspections of how similar the predicted and observed trends are across conditions, as it has been argued that visual inspection is the best way to assess the validity of Bayesian models (Gelman, Carlin, Stern, & Rubin, 2003; Gelman & Shalizi, 2013).

We also analyze the performance of the model by assessing whether key effects predicted by the model to occur across conditions materialize in participants’ observed behavior. We test whether the observed and predicted trends agree in terms of direction and statistical significance, as well as whether the two trends statistically significantly differ from each other. Ideally, we will find that both the predicted and observed trends agree in terms of direction and significance, and are not statistically significantly different from each other. Some discrepancies would indicate a degree of inaccuracy, but would not be too problematic: for example, if we find a statistically significant trend occurring in the same direction for both predictions and observations, but one is statistically significantly stronger than the other, at least the qualitative prediction about the trend is the same. Conversely, if we find a trend is statistically significant for predictions but not observations (or vice versa), but the two trends are themselves not statistically significantly different from each other, the discrepancy may be due only to random error. The most problematic discrepancies would be when the predicted and observed trends go in opposing directions and are both statistically significant, or are statistically different from each other with one effect significant and the other null.

In Study 1, we measure belief updating about hypotheses in response to potentially biased sources’ testimony; this study combines data from two virtually identical versions of the study for the sake of maximizing statistical power. In Study 2, which was preregistered, we conceptually replicate Study 1, and introduce new tests of the model's predictiveness for inferences about source characteristics. The Supplementary Materials contain a “Supplementary Study” (Supplementary Material 3), which explores inferences about source characteristics, returning corroborating results; while we think this study's results are trustworthy, some methodological issues led us to relegate it to the supplement.

All studies took place online through the survey participation platform Prolific, using British participants. They were programmed in Qualtrics and data analyzed in *R* (R Core Team, 2025). We used the *R* package gRain (Højsgaard et al., 2012) to generate predictions from the model. Unlike Harris et al. (2016), but like Madsen (2019), we assess the performance of the model with no free parameters—we enforce that all priors and likelihoods for the generation of predictions are either taken from participants’ responses or assumed; there is no parameter‐fitting.^4^ Scripts and data for all studies are available via our OSF project for this article.

## 2 Study 1

The aim of Study 1 was to test whether our Bayesian Network model could accurately predict how people would update their beliefs in response to claims made by sources who differ in terms of their apparent bias and expertise. We have combined results from two experiments whose designs were identical but for minor changes to the wording of some questions and scale labels, which we have verified have no impact on the results (see Supplementary Material 2). Combining the studies, therefore, maximizes statistical power.

### 2.1 Method

#### 2.1.1 Participants

We recruited 224 participants, but excluded 79, leaving a final sample of *N* = 145 (11 failed at least one of four attention checks, 68 failed at least one of two memory checks—details below). The final sample consisted of 72 men, 72 women, and 1 agender person; their median age was 35 with a range of 19−74. We also asked for participants to describe their ethnicity using the UK census options: 108 selected “English, Welsh, Scottish, Northern Irish, or British,” 13 “Any other White background,” 5 Irish, 6 African, 5 Pakistani, 1 “Any other Asian background,” 1 “Any other Black, African or Caribbean background,” 1 “Gypsy or Irish Traveller,” 1 Indian, 1 “Mixed or Multiple ethnicities,” 1 “White and Asian,” 1 “White and Black African,” and 1 “White and Black Caribbean.”

The study was advertised to pay £7.50/h, with a 10p bonus for passing memory checks. We informed participants that the study had undergone the review procedure of the Department of Psychology Research Ethics Committee at the University of Cambridge before they provided consent.

#### 2.1.2 Power analysis

A post‐hoc sensitivity power analysis suggests this study is well‐powered. With six observations per participant, we have 870 observations in total, which affords 80% power to detect effects as small as *r* = .095 (G*Power 3.1.9.7, bivariate normal correlation, two‐tailed, alpha = .05, *r* ≥ ρ, ρ H0 = 0). This tells us that we can be reasonably confident that if we obtain a null result, the population‐level correlation is either actually null or smaller than 0.095.

#### 2.1.3 Design

Participants were shown six fictional dialogues between two characters—James and Anne—concerning the effectiveness of new policies their Government had implemented. In each dialogue, James told Anne what a source had said about the effectiveness of the policy, and then participants were asked to estimate what Anne's degree of belief that the policy had benefited the country should be.

We varied the details of the source and their testimony with a 3 x 2 x 2 within‐subjects manipulation: Source Bias (Pro‐Government, Neutral, Anti‐Government) x Source Expertise (High, Low) x Source Testimony (Pro, Anti). To effectuate the Source Testimony manipulation, in the Pro condition, James told Anne the source had said the policy was “going to be really beneficial for the country,” and in the Anti condition, that it was “going to be really harmful for the country.” The descriptions used for the sources to create the Source Bias x Source Expertise manipulations are shown in Table 2. We only showed participants one claim from each source to avoid confusion and order effects, meaning each completed six trials. To achieve this within the design constraints, we randomized participants between two “streams”—A and B—where the sources provided different testimony, as shown in Table 2.

We chose “a construction worker” and “the head of a policy research institute” as exemplars of people with comparatively low and high expertise on policy issues, following a pilot study into the perceived policy expertise of people in various professions. The pilot showed that the heads of policy research institutes were perceived to have more expertise on policy issues than politicians, political journalists, economists, and elite businesspeople, as well as construction workers.

#### 2.1.4 Procedure

After obtaining consent, we provided task instructions. We told participants they would be asked questions about political policies in an imaginary Western democracy, which was not the UK or USA. This was to dissuade participants from making assumptions about the ideological leaning of the government, which could have affected their responses, but while still applying their general beliefs about how source bias manifests in politics. We told them there were only two parties in this country, which we would call “the Government party” and “the Opposition party.” We also told participants that we would use checks to monitor whether they were paying attention throughout the survey.

The main experiment then consisted of three sections: Priors, Dialogues, and Conditionals, which were presented in that order. In the Priors section, we asked participants to provide their priors for the sources’ characteristics. They were given the descriptions of the sources from Table 1, and asked to estimate their Leaning, Bias Intensity, and Expertise, in that order. These judgments were blocked by source, giving six blocks, and the blocks were presented in a random order, each on a separate page,^5^ with an attention check block embedded as well—for details, see “Attention and Memory Checks” below.

Then came the Dialogues section. At the start, we informed participants to treat all the dialogues separately, and to not use any conclusions drawn from one dialogue to affect how they interpreted another. We also instructed them “You can assume everything James says is true.” Each dialogue had the same script, which was, with original emphases:

James: Do you have any opinion on the government's new {**x**} policy?
Anne: No, I have no idea whether it's good or bad.
James: Well I heard it's going to be really {**beneficial/damaging**} for the country.
Anne: Where did you hear that?
James: I heard from {**source**}.

The policy area, x, was randomly selected, using a Latin square (see Supplementary Material 4), for each participant from one of the following: Agricultural, Housing, Healthcare, Transport, Foreign Trade, Energy. The choice of “beneficial” or “damaging” depended upon the Source Testimony condition (Pro vs. Anti, respectively), and the source description depended upon the Bias Direction and Expertise condition. After each dialogue, we measured the participant's estimate for Anne's degree of belief that the policy would benefit the country. Every participant completed six dialogues—one for each source—in a random order.

We also gave participants a dummy dialogue trial, which resembled the other trials and always came at the start of the Dialogues section. This was not a real trial—participants were not asked to estimate Anne's degree of belief—but served as a practice memory check in order to impress upon participants the need to carefully attend to and remember the key bits of presented information, and alerted them to the fact that further “real” memory checks would follow—see “Attention and Memory Checks” below. We presented two further memory check questions within the Dialogues section—see “Attention and Memory Checks” below.

Then came the final “Conditionals” section, where participants provided conditional probability estimates for the quantities in Table 2. An attention check, which asked participants to give a particular numerical response, was embedded within this block at a random point—see “Attention and Memory Checks” below. After completing this section, we asked for demographic information, then debriefed and thanked participants.

**Table 2:** Descriptions of sources in Study 1

			Testimony	
Expertise	Bias	Description	Stream A	Stream B
High	Anti‐Gov.	“the head of a policy research institute who normally always support the Opposition party”	Pro	Anti
High	Pro‐Gov.	“the head of a policy research institute who normally always support the Government party”	Anti	Pro
High	Neutral	“the head of a politically‐neutral policy research institute”	Pro	Anti
Low	Anti‐Gov.	“a construction worker who normally always supports the Opposition party”	Anti	Pro
Low	Pro‐Gov.	“a construction worker who normally always supports the Government party”	Pro	Anti
Low	Neutral	“a construction worker who sometimes supports the Government party and sometimes supports the Opposition party”	Anti	Pro

#### 2.1.5 Attention and memory checks

##### 2.1.5.1 Memory checks

A dummy memory check block was presented immediately after the task instructions for the Dialogues section of the experiment. Participants were shown a dialogue where James stated he had heard an Opposition politician say that the government's new banking policy was going to be beneficial for the country. On the following page, where the dialogue was no longer visible, participants were given a multiple‐choice question and asked to indicate whether James had heard about the policy from a “Government politician,” “Opposition politician,” or “A construction worker.” On a second page, they were given another multiple‐choice question, and had to indicate whether that person had said the policy would be “Beneficial” or “Harmful.” After completing both questions, participants were informed that these memory checks were “just for practice.” They were then informed that similar tests would be carried out later on, and that if their answer to one randomly selected test question was correct, they would win a 10p bonus.

Two further memory checks were served after a dialogue was presented. The first memory check question was served following the High Expertise, Anti‐Government Bias, Pro‐Government Testimony trial if the participant was in Stream A, or the High Expertise, Anti‐Government Bias, Anti‐Government Testimony trial if they were in Stream B. This question asked participants which source James had heard about the policy from in the preceding dialogue, and gave participants the following multiple‐choice options: “The head of a policy research institute who normally always support the Government party,” “The head of a policy research institute who normally always support the Opposition party,” or “A construction worker.”

The second memory check question was served after the Low Expertise, Pro‐Government Bias, Pro‐Government Testimony trial if the participant was in Stream A, or the Low Expertise, Anti‐Government Bias, Pro‐Government Testimony trial in Stream B. This question asked participants what the source had said the impact of the policy would be in the preceding dialogue, giving them the following multiple‐choice options: “Harmful,” “Beneficial.”

Notably, whichever stream participants were in, the direction of the source's bias was consistent with their testimony for one check, but inconsistent on the other.

##### 2.1.5.2 Attention checks

One block within the Priors section consisted of three attention check questions. This block closely resembled the other blocks in the section, which also contained three questions each, and the scales were labeled in the same way as the “real” questions; however, the text of the attention check questions just asked participants to give an answer of 20 on the slider. Therefore, participants had to answer three concurrent attention checks, which required the same response; this was done so that inattentive participants would not easily notice that these questions were of a different format to the adjacent “real” questions.

A fourth attention check was included within the Conditionals section. In this section, all questions were presented on their own page, so this attention check question was as well; it began with a preamble which resembled the other questions so as not to make it immediately obvious to nonattentive participants, but then unambiguously morphed into an attention check: “If an **impartial expert** was asked whether the Government's new policy would be beneficial or harmful to the country. It doesn't matter, please just put an answer of 73 to this question so that we know you are paying attention.”

To avoid exclusion, participants had to pass all four attention checks and both of the “real” memory checks.

#### 2.1.6 Measures

All measurements were made on 0−100 slider scales, which began with a default position of 50. The value that corresponded to the slider's position was displayed immediately above it. The question used to measure estimates of Anne's belief in the policy's effectiveness was the same for both studies: we asked participants below each dialogue (but on the same page), “In light of the dialogue above, what do you think Anne's opinion should now be about the {x} policy?”, where *x* was the corresponding policy. The slider was labeled “They should be totally confident it's harmful” at 0, “They should be unsure” at 50, and “They should be totally confident it's beneficial” at 100.

The questions used to measure source characteristics were slightly different in the two versions (A and B) of the experiment, which compromised Study 1.

##### 2.1.6.1 Version A

To measure Bias Intensity, participants were given the corresponding description from Table 2, and asked to imagine they were hearing them talk about policy issues. They were then asked “When it comes to disagreements between the Government party and the Opposition party, how strongly do you think they would be biased towards one political side?” and given the further information, “The stronger their bias, the more likely it is they would make false claims that support their political side.” The slider was labeled “Not biased at all” at 0, and “Totally biased” at 100. For Leaning, they were asked “If they are biased towards one political side, which side is that?”; the slider was labeled “Against the government party” at 0 and “Towards the government party” at 100. For Expertise, they were then asked, “When this source talks about political issues that they are neutral towards, i.e. not biased in any way, how accurate would they be?”; the slider was labeled “What they say is just as likely to be true as false” at 0, and “They would be totally accurate” at 100.

##### 2.1.6.2 Version B

For Bias Intensity, we asked “Imagine you were hearing {source description} talk about policy issues. Which of the following is closest to the truth?” 100 = “This person is biased when discussing policy issues” 0 = “This person is impartial when discussing policy issues,” with the slider labeled “0 = Impartial” and “100 = Biased.” For Source Leaning, we asked “And which of the following is closest to the truth?” 100 = “If this person was biased, they would be biased towards the government party” 0 = “If this person was biased, they would be biased towards the opposition party.” Remember, the person is a {source description}, with the slider labeled “0 = Opposition,” “50 = Neither,” and “100 = Government.” For Expertise, we asked “And which of the following is closest to the truth?” 100 = “If they could avoid being biased, this person would be an expert on policy issues” 0 = “Even if they could avoid being biased, this person would still not be an expert on policy issues.” Remember, the person is {source description}, with the slider labeled “0 = Not an expert” and “100 = Expert.”

### 2.2 Results

**Fig. 3:**
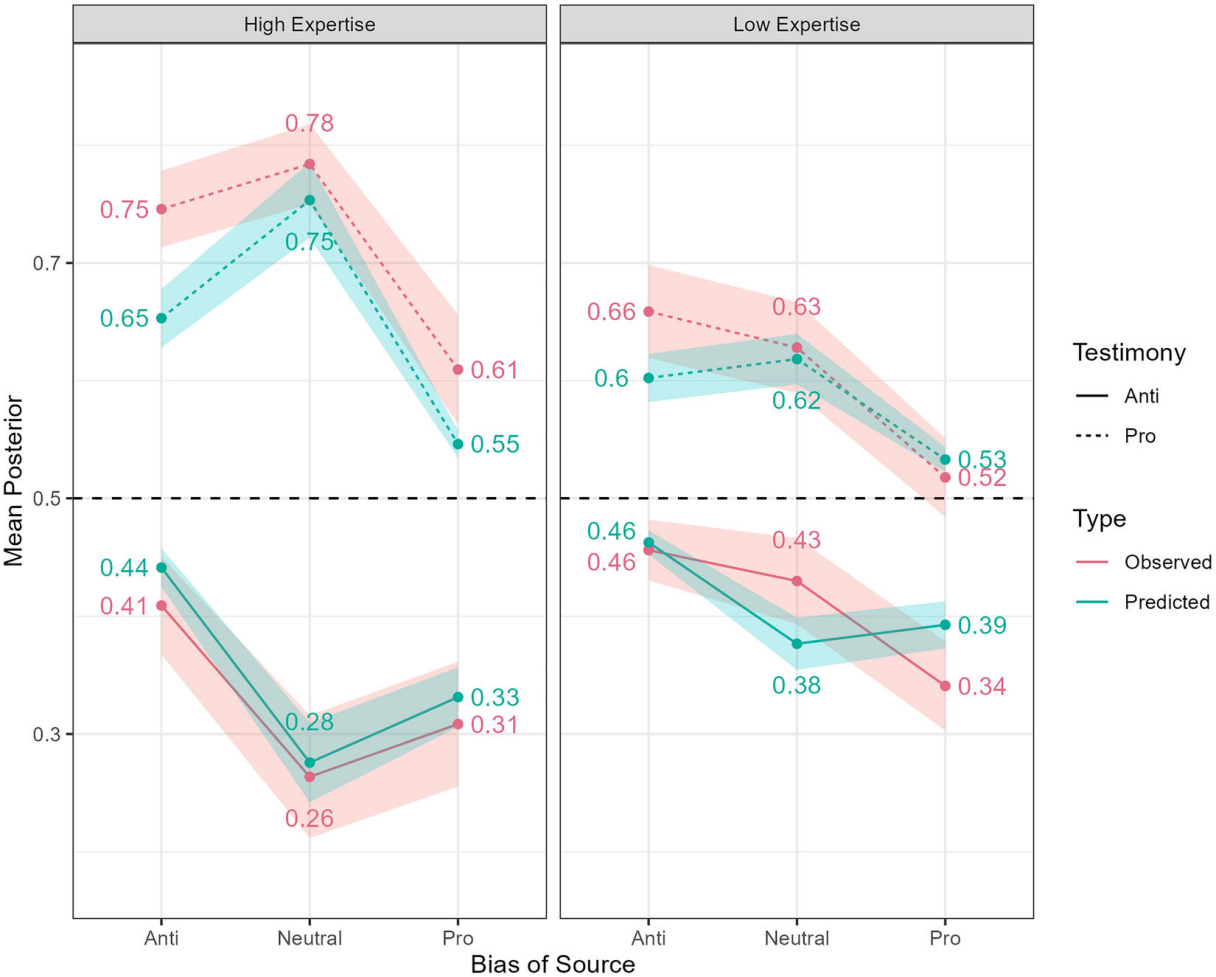
Mean predicted and observed posteriors, across conditions, with 95% confidence intervals.

We used the Bias‐and‐Expertise Model to predict the posterior degrees of belief each participant would assign to Anne in response to each piece of source testimony. We assumed their priors for the effectiveness of the policy would be 0.5, as Anne had said she had “no idea” about them. We then compared these predicted posteriors to the observed posteriors that participants estimated. Fig. 3A shows the mean predicted and observed posteriors, with 95% confidence intervals, across all 12 conditions, for Study 1a; Fig. 3B does the same for Study 1b.

#### 2.2.1 Visual inspection

From visual inspection, the predictions provide a broadly good fit, though not a perfect one. The largest difference between an observed and predicted mean for a given condition is 0.10, and in 9/12 cases, the difference is 0.05 or less; in 7/12 cases, the predicted mean is within the 95% confidence interval of the observed mean. Comparing adjacent conditions, there are two cases of nonparallel trends when comparing observations and predictions (out of a possible eight): for Low Expertise sources with Anti‐Government Testimony, the model predicts a 0.01 higher mean for sources with a Pro‐Government versus Neutral leaning (independent‐samples *t*(143) = 1.07, *p* = .287), but the observed difference is 0.09 points lower (independent‐samples *t*(143) = 3.38, *p* < .001); for Low Expertise sources with Pro‐Government Testimony, the model predicts a 0.02 lower mean for sources with an Anti‐Government versus Neutral leaning (independent‐samples *t*(136) = 1.08, *p* = .280), but the observed difference is 0.03 higher (independent‐samples *t*(136) = 1.10, *p* = .271). Both these discrepancies then reflect cases where the participants regarded a source whose testimony conflicted with the direction of their bias as slightly more persuasive than a neutral source, though the model predicted they should have found the neutral source more persuasive. However, given that neither predicted difference is statistically significant, and only one of the observed effects is, and furthermore that this same discrepancy is not seen for the High Expertise sources in any case, it seems more likely these discrepancies reflect noisiness in the model's predictions rather than a systematic error in the model.

#### 2.2.2 Comparison of trends

**Fig. 4:**
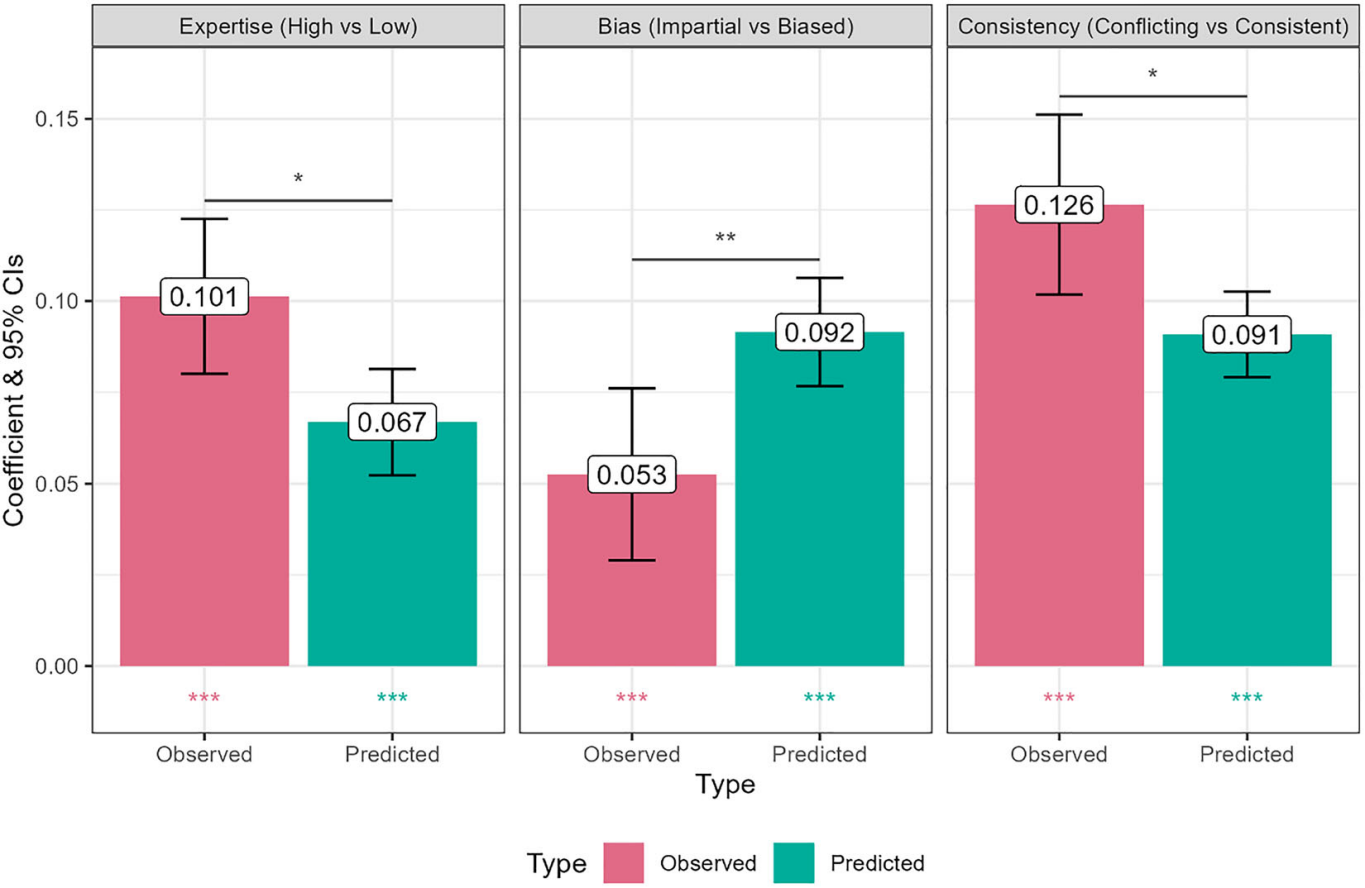
Predicted and Observed trends caused by our manipulations, and whether the trends differ (significance stars). Colored stars indicate whether the corresponding contrast is statistically significant.

We analyzed three key trends caused by our manipulations, looking at the predicted trend, the observed trend, and calculating an interaction term when pooling both predictions and observations to ascertain whether the predicted and observed trends differ. From Fig. 3A, we can see the model naively predicts that under conditions like this experiment, three main effects should affect how much people are persuaded by the source's testimony: higher expertise should lead to greater persuasion, being neutral (low bias intensity) should lead to greater persuasion than being biased (collapsing across directions), and among biased sources, having a leaning which is *conflicting* with the testimony (i.e., a pro‐Government leaning but anti‐Government testimony, or anti‐Government leaning but pro‐Government testimony) should lead to greater persuasion than having a leaning *consistent* with the direction of the testimony (both pro‐Government or both anti‐Government). To test for these trends, we constructed a measure of “Persuasion” by finding the difference between the observed/predicted posterior and 0.5 in the direction of the testimony (pro‐Government testimony: Posterior − 0.5, anti‐Government testimony: 0.5 − Posterior). We then conducted multi‐level regressions, which included the key contrast of interest for each trend as a fixed‐effects predictor (Expertise: High vs. Low; Bias: Neutral vs. Biased; Bias Consistency: Conflicting vs. Consistent [with Neutral participants excluded]), and random intercepts for Participant ID and the Testimony. We conducted these regressions separately for the predicted posteriors and the observed posteriors. We then conducted another regression pooling both the predicted and observed posteriors, and adding in a fixed‐effects interaction between “Type” (Predicted vs. Observed) and the contrast, as well as a fixed‐effects main effect of Type. Fig. 4 shows the Observed and Predicted trends, as well as the significance of the interaction term. In all cases, all individual contrasts are significant (*p*s < .025), and the predicted and observed trends go in the same direction, but all the interactions are significant too (*p*s < .017). Thus, while the model makes accurate predictions about the occurrence and direction of key trends, its estimates of their magnitude are consistently significantly different—underestimating the effect of Expertise by 0.034 points, overestimating the effect of Neutrality by 0.039 points, and underestimating the effect of there being Conflict versus Consistency between a source's leaning and Testimony by 0.035 points.

#### 2.2.3 Statistical measures of model performance

As for statistical measures of model performance, the predicted posteriors correlated significantly with the observed posteriors, *r*(868) = .609, *p* < .001, with a Brier score (Mean Squared Error) of 0.036, and VM of 95.3%. Technically, since we obtain multiple observations and predictions per participant, the independence criterion is violated; we, therefore, also calculated the correlation for each individual between their observations and predictions, then found the mean. The violation appears not to have mattered in practice, as the mean individual correlation was slightly larger, at *r* = .677 (the downside to using this approach is that we cannot calculate a *p*‐value).

##### 2.2.3.1 Individual differences

Calculating the individual‐level correlations affords some analysis of individual differences. For three participants, a correlation coefficient could not be calculated because they provided an estimated posterior of 0.5 in every condition; eight participants had negative correlations, none had correlations between zero and 0.1, three between 0.1 and 0.3 (small), 17 between 0.3 and 0.5 (moderate), and 114 above 0.5 (large). We performed regressions to ascertain if the size of a person's correlated was affected by demographic factors, but found no relation to Ethnicity (*R* ^2^ = 2.5%, *p* = .993), Gender (*R* ^2^ = 14.4%, *p* = .123), Age (*R* ^2^ = 1.3%, *p* = .174), or their self‐reported position on a 7‐point left‐right ideology scale (*R* ^2^ = .0%, *p* = .810).

### 2.3 Discussion

Study 1 shows that the Bias‐and‐Expertise model is effective at predicting belief updating in response to testimony from potentially‐biased sources. Relative to Harris et al.’s Trustworthiness‐and‐Expertise model (2016), the percentage of variance in mean posteriors across conditions explained by our predictions compares well: 95% against 80−90%. We also make individual‐level predictions, finding correlations that are significant and large in size. On the 100‐point response scale used, predicted mean positions in response to a given source were always within 10 points, and usually within 5.

Importantly, Study 1 returned a trend that is incompatible with the Trustworthiness‐and‐Expertise model and the Reliability model. As the “Consistency” panel in Fig. 4 shows, participants were more persuaded by sources’ testimony when it conflicted with their bias direction, as predicted. This pattern of updating is incompatible with competing models, because they contain no information about the leanings of sources or hypotheses. In these models, sources simply have a latent probability of providing false testimony, and that probability applies to whatever the testimony happens to be. It is impossible for agents using the model to be *persuaded* a different amount depending on what their source's testimony is.

Therefore, Study 1 not only returns evidence of the model's validity, which is of a similar type and quality of previous evidence for competitor models, it in fact returns evidence that shows the superiority of this model for applications to political testimony. That said, there are some discrepancies in our evidence and limitations to this study. There were a couple of instances of nonparallel trends, though, as noted, in both cases, the predicted trend was itself nonsignificant, and the discrepancies remain small in absolute terms.

The design for this study has a number of notable limitations. Since our dialogues all featured the same characters, what was learnt about them in one scenario could have affected participants’ assumptions about them for later scenarios. Another potential carry‐over issue could arise because participants were given information about the effectiveness of different government policies, which could have affected their assumptions about what Anne's beliefs about the effectiveness of other policies in similar areas might be. To try to avoid this latter problem, we purposefully picked relatively orthogonal policy areas where good performance in one area would not necessarily imply that the government was likely to perform well in another (Agricultural, Housing, Healthcare, Transport, Foreign Trade, Energy). To more broadly try to avoid carry‐over effects, we explicitly instructed participants: “treat all the dialogues separately ‐ do not try to use any conclusions you draw from one dialogue to affect how you interpret another dialogue.” However, we cannot guarantee compliance with this instruction.

**Fig. 5:**
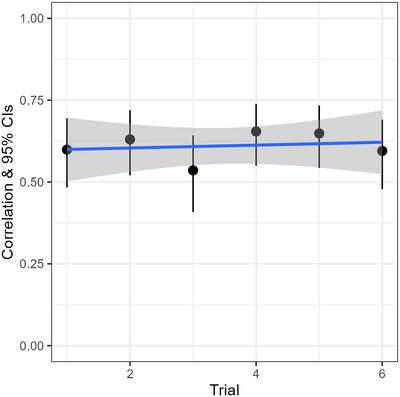
The correlation between observed and predicted posteriors was stable, and did not weaken for later trials, implying no interference from carry‐over effects.

Ultimately, if there are carry‐over effects which affect the predictiveness of the model, this should manifest as a trend whereby the correlation between predicted and observed posteriors weakens for trials that participants completed later, as the model treats all dialogues as completely independent. As Fig. 5 shows, there was no such weakening over time—trials that participants completed earlier in the block had just as strong a correlation as trials completed later. Therefore, carry‐over effects do not seem to have caused any issues in Study 1.

It is also worth considering whether it was plausible to use a construction worker as our exemplar of a low‐expertise source. We mean no disrespect to construction workers, many of whom we are sure take an interest in and are knowledgeable about politics. However, in contrast to someone who is the Head of a Policy Research Institute, we considered, and our pilot data confirmed, that a construction worker would be someone who most participants would think was likely to be less knowledgeable. Is it plausible that James would have heard testimony about the effectiveness of political issues from a construction worker? Given that James does not disclose how or where they heard about the source's opinion on each policy, we see no reason why not—in the UK, it is quite common for the opinions of “ordinary” people to be solicited and shared on popular political TV shows, like Question Time, or as “vox pops” on News programs, so participants might have thought James had encountered the testimony there, which is also the most likely place where James would have heard the opinion of someone they knew to be the Head of a Policy Research Institute.

We should also consider the possible impact of our high exclusion rate. We excluded 30% of our sample for failing memory checks, and an additional 5% for failing attention checks. The memory checks were implemented to ensure participants attended to and retained the important pieces of information from each dialogue, and reasoned about them in order to answer the questions, rather than just reading off information when answering the questions. We consider that this approach gives better insights into people's reasoning. We also considered that in the real world, when reasoning about how to update their beliefs in response to potentially biased sources like politicians, media commentators, or fellow citizens, people will often have a fairly clear idea of the source's level of bias and expertise in their minds, which conditions their response; yet, this is difficult to fabricate in an artificial experimental setting, particularly when we want to gather responses to multiple different kinds of source in quick succession. We think that incentivizing participants to remember the crucial pieces of information presented about each source—and excluding those who did not appear able to—gets us closer to the real‐world scenario, and, therefore, improves generalizability. However, by restricting our sample to only the most attentive participants, we may also lose some generalizability, as our results may only be true for particularly careful and attentive participants. The easiest way to establish the impact of the memory checks is to re‐analyze the data when those who fail the checks are allowed to remain in the dataset. This boosts the sample size to *N* = 213, and most statistical measures of performance are worse, but not by much. The correlation between observations and predictions falls to .54, the mean individual correlation falls to 0.59, the Brier Score (MSE) rises to 0.039, and the VM actually increases to 95.6% (which is very slightly better). This suggests the model still works well even when we are less rigorous about the level of engagement shown by participants, but that greater engagement does help increase predictability.

## 3 Study 2

### 3.1 Introduction

Study 1 tested how well the model performs in predicting how participants update their beliefs about hypotheses in response to source testimony, given their perceptions of those source's characteristics. But the model also makes predictions about the inverse process—how people update their perceptions in response to source testimony, given their beliefs about the hypothesis at hand. Collins et al. (2018) have demonstrated that their Bayesian Network utilizing the source characteristic “Reliability” performs “bi‐directionally,” with people ascribing lower reliability to sources who make claims they find implausible, as well as updating less in response to sources who are deemed unreliable. Does the Bias‐and‐Expertise Model also perform bi‐directionally? The purpose of Study 2 was to address this by testing how well the model performs in predicting updating about source characteristics, in addition to hypotheses. We adapt the scenario in Study 1, by giving participants evidence about the effectiveness of the government's policies, so that they can infer bias and (in)expertise when sources’ claims conflict with the evidence. We then test how the characteristics inferred from hearing the source's claim about one policy influence how people respond to their claims about a second policy later on.

There were also some cosmetic changes—we swapped the characters’ genders so that “Rob” was provided with information about the policies by “Jen,” and we displayed cartoon visualizations of the dialogues rather than text descriptions, which we anticipated might result in stronger treatment by making the information clearer. We also collected a larger number of participants, who only completed one trial each, to allay any concerns caused by the repeated‐measures design. The study's design, analysis, and exclusion criteria were preregistered; the preregistration is available in our OSF project for this article.

### 3.2 Method

#### 3.2.1 Participants

We recruited 757 participants, who were paid £7.50/h. We excluded 33 participants in total, in accordance with our preregistered exclusion criteria (32 failed at least one of two comprehension checks, 1 failed the single attention check), leaving a final total sample of *N* = 724. The sample consisted of 356 men, 362 women, 4 nonbinary people, and 2 who preferred not to say. Using the British Government's standard options for classifying race/ethnicity, 585 described their ethnic background as “English, Welsh, Scottish, Northern Irish, or British,” 37 as “Any Other White,” 15 as Indian, 11 “White and Asian,” 11 “Any Other Asian,” 10 African, 9 Pakistani, 8 “White and Black Caribbean,” 8 Irish, 8 Chinese, 8 Caribbean, 6 “Mixed or Multiple Ethnicities,” 4 Bangladeshi, 3 “Any Other Ethnicity,” 2 “White and Black African,” and 1 Arab. The median age was 37 with a range of 18−81. We informed participants that the study had undergone the review procedure of the Department of Psychology Research Ethics Committee at the University of Cambridge before they provided consent.

This study was adequately powered by conventional standards, with 724 participants providing one observation each; we have 80% power to detect effects as small as *r* = .15 (G*Power 3.1.9.4 sensitivity test, independent‐samples Pearson's correlation, two‐tailed, alpha = 0.05).

#### 3.2.2 Design

In this study, we present participants with a scenario where one character (Jen) gives another (Rob) evidence about whether a new Government policy (regarding tax) has been beneficial or harmful, and tells them what a media source had said about its impact, after which we measure source perceptions. We employ a 5 x 2 between‐subjects manipulation of the strength and direction of this evidence (strong harmful: “strong evidence it has damaged the economy,” weak harmful: “some evidence it has damaged the economy but they couldn't be certain,” neutral: “no evidence either way as to whether it has boosted or damaged the economy,” weak beneficial: “some evidence it has boosted the economy but they couldn't be certain,” strong beneficial: “strong evidence it has boosted the economy”) and the testimony (harmful vs. beneficial).

Additionally, Jen tells Rob what the same media source said about a second policy for which no evidence is given (the “critical” policy), and we measure their prior and posterior for that policy's impact. This second piece of testimony was manipulated independently of the first, meaning that posteriors for the second policy's impact were subject to a 5 x 2 x 2 between‐subjects manipulation overall. The interaction between the evidence and the first piece of testimony should influence participants’ perceptions of the source's bias and expertise. These perceptions should, in turn, influence how they update their beliefs about the second claim.

#### 3.2.3 Procedure

After opting to take part and providing informed consent, we provided participants with task instructions and information about the experimental scenario, which, as before, involved telling them that two people who lived in a fictional Western democracy were discussing politics, that they could trust Jen's information, and that there would be attention checks.

Then, the experimental trial began. The trial was spread across five pages. On page 1, Jen asked Rob if he had any opinion about the Government's new tax policy, to which he responded he knew “nothing about it.” Then Jen told Rob that she had seen “a report put together by a group of neutral experts” which had said about the policy had boosted or damaged the economy, depending on the condition; Rob said he “hadn't heard about that.” Beneath, we measured participants’ judgment of what Rob's belief about whether the tax policy had boosted the economy should be. While this is a posterior with respect to the evidence, it is a prior for the subsequent judgments, so we refer to it as the prior for the tax policy. Below this question were two multiple‐choice comprehension questions that asked participants to identify the policy area and what the experts had found.

On page 2, Jen asked Rob whether he reads the newspaper “The Enquirer”; Rob replied that he did not and had not even heard of it. This was a treatment designed to enforce neutral priors for the source's characteristics. Below this, we measured people's judgments of what Rob's beliefs about the Enquirer's Bias Intensity, Source Leaning,^6^ and Expertise would be, in that order. Below these questions was an attention check, which asked people to give an answer of 92 on a slider.

On page 3, Jen showed Rob the front page of The Enquirer, which said the tax policy had either boosted or damaged the economy. Below the newspaper, we reminded participants of what Jen had said the team of experts had found, then measured their posterior judgments of the Enquirer's Bias Intensity, Leaning, and Expertise, in that order.

On page 4, Jen asked Rob if he had any thoughts on the Government's new crime policy, to which he replied, “I don't know anything about it!”. Below, we measured participants’ judgment of Rob's belief that the tax policy had reduced the crime rate.

On page 5, Jen showed Rob another front page from the Enquirer where they claimed the crime policy had either reduced or increased the crime rate. Beneath, we measured participants’ posterior judgment of Rob's belief that the tax policy had reduced the crime rate. Participants then provided demographic information, were thanked, debriefed, and redirected back to Prolific.

#### 3.2.4 Stimuli

The cartoons consisted of a Caucasian man and woman facing forward, with speech bubbles. Both had identical smiling expressions and wore casual clothing. In the “Beneficial” testimony condition for the tax policy, the headline on the front page of the newspaper was “Economic Miracle” with the subheadline “Economy soars following tax reforms,” and an image of an increasing bar chart and a green arrow trending upward. In the “Harmful” testimony condition, the headline was “Economic Disaster” with the subheadline “Economy crashes following tax reforms,” and an image of a red line graph trending downward. For the crime policy, the headline was “Crime in Retreat” in the “Beneficial” testimony condition, with the subheadline “Crime rate plummets in wake of reforms,” with an image of a red line trending downward on a graph (which was different from the one that accompanied the harmful tax policy headline). In the “Harmful” testimony condition, the headline was “Crime on the Rise,” with the subheadline “Crime rate skyrockets in wake of reforms,” with an image of a cross formed by two lengths of yellow crime‐scene tape that said “Crime Scene.”

#### 3.2.5 Measures

As before, all measurements were made on 0−100 slider scales, which began with a default position of 50. The value that corresponded to the slider's position was displayed immediately above it. To measure beliefs, we asked “On a scale of 0‐100, how confident do you believe Rob is that government's new {crime/tax} policy has {reduced the crime rate/boosted the economy}?”, with the slider labeled “0 = Totally confident it has not,” “50 = Totally unsure,” and “100 = Totally confident it has.” For Bias Intensity, we asked “Do you think Rob believes The Enquirer is biased or impartial when it comes to covering government policy, and how confident is he?” with the slider labeled “0 = Completely certain they are impartial,” “50 = Completely unsure,” and “100 = Completely certain they are biased.” For Source Leaning, we asked “And to the extent Rob believes The Enquirer is biased at all, do you think he believes it is bias towards or against the government, and how certain is he?”, with labels “0 = Definitely against the government,” “50 = Completely unsure,” and “100 = Definitely towards the government.” For Expertise, we asked, “And what if The Enquirer wasn't biased, would it then be an expert source when it comes to government policy, or would it still not be an expert source? Think about what Rob believes and how confident he is,” with labels “0 = Certain it would not be an expert source,” “50 = Completely unsure,” and “100 = Certain it would be an expert source.”

#### 3.2.6 Hypotheses

We preregistered that the posterior ratings for Bias Intensity, Source Leaning,^7^ Expertise, and the effectiveness of the crime policy would correlate significantly with the predicted posteriors. We also preregistered to calculate Brier scores and VMs as in previous studies.

### 3.3 Results

As preregistered, we generated predicted posterior source perceptions for each participant by inputting their prior for the effectiveness of the tax policy (the first issue) and their prior source perceptions into the model, then updating the model to the source's testimony about the policy. We compare these predictions against the observed posterior source perceptions. To generate the predicted posteriors for the effectiveness of the crime policy (the second issue), we used the observed posterior source perceptions and prior judgment of the crime policy's effectiveness as priors, then updated to the source's testimony about the crime policy.

**Fig. 6:**
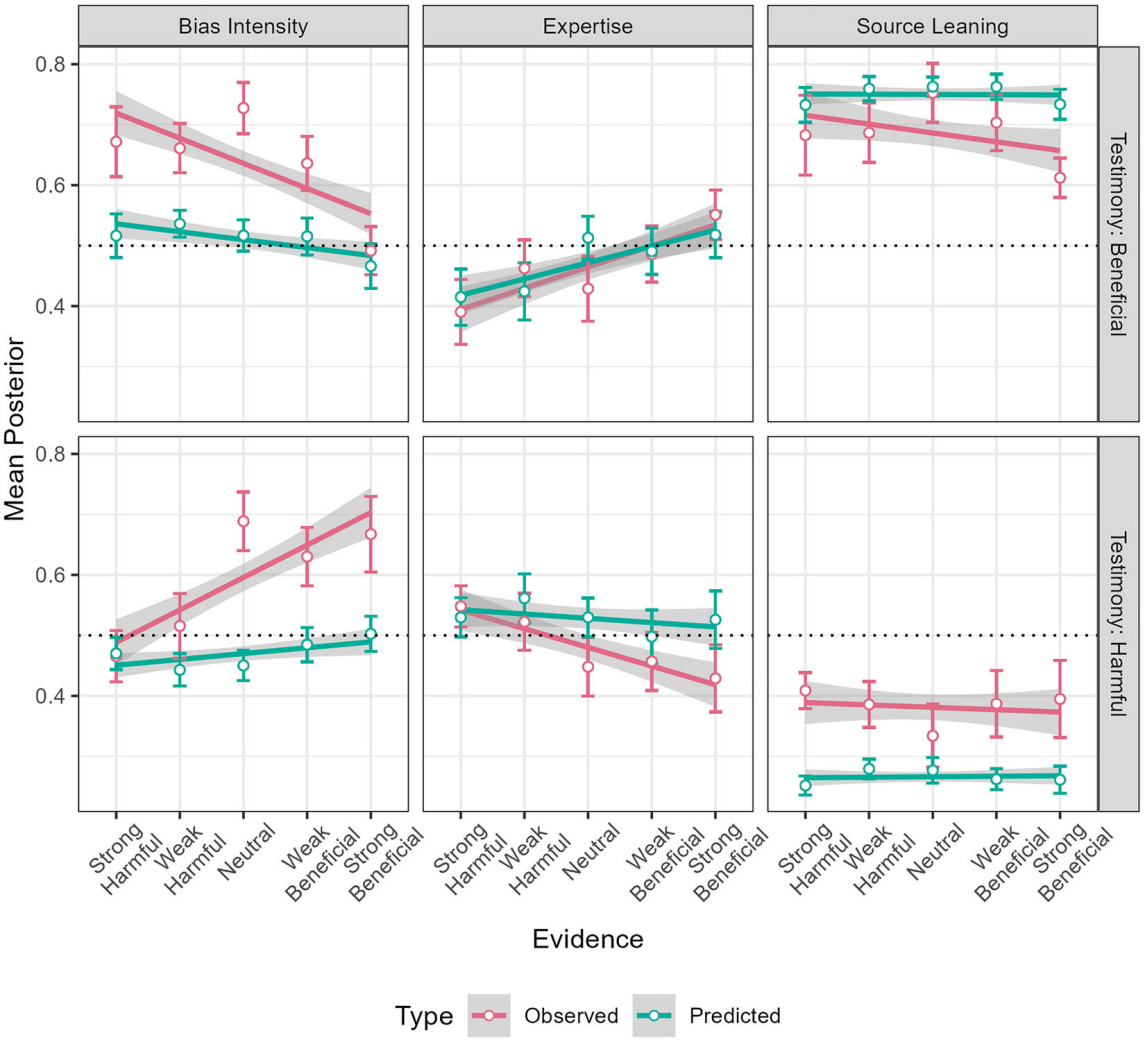
Regression lines for predicted and observed posterior source perceptions across conditions, with means and 95% confidence intervals.

**Fig. 7:**
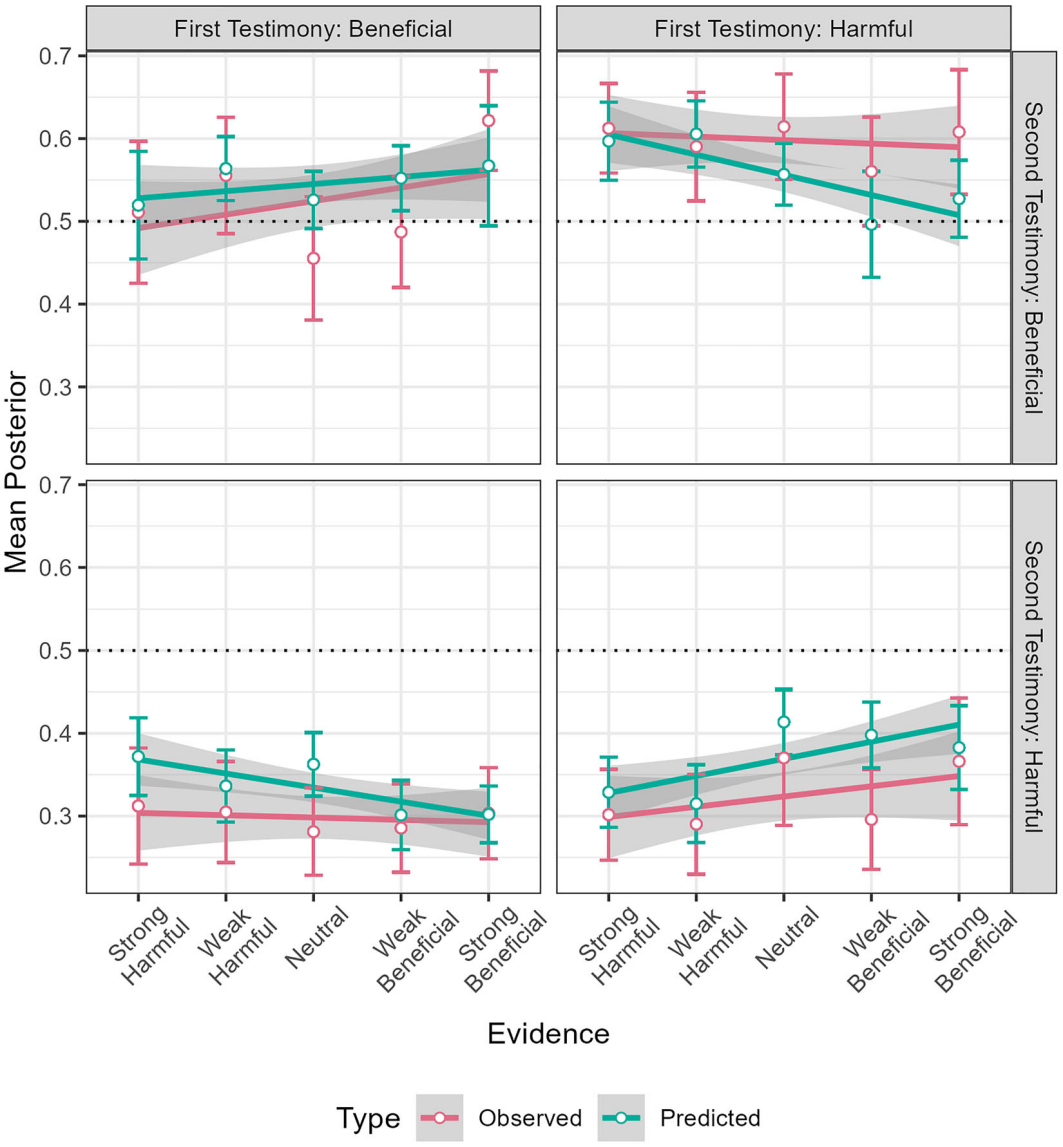
Regression lines for predicted and observed posteriors for the crime policy across conditions, with means and 95% confidence intervals.

Fig. 6 shows the regression lines for predicted and observed posteriors across conditions, with means and 95% confidence intervals, for the source perceptions. From visual inspection, observed and predicted trends are generally qualitatively similar, going mostly in the same direction. Posterior judgments of Bias Intensity are, on average, stronger than predicted, as well as more sensitive to the evidence presented. For Expertise, trends are very similar, though less so for “harmful” testimony. For Source Leaning, the predictions are too extreme. One discrepancy is that for both kinds of Testimony, Neutral sources elicit higher observed Bias Intensity posteriors than expected from the regression line for observed Bias Intensity posteriors, by ∼0.10. Fig. 7 shows the predicted and observed posteriors for Hypothesis updating for the second (crime) policy, with close trends.

As hypothesized, our predictions significantly correlated with the observations for each variable. For Bias Intensity, the correlation was *r*(722) = .16, *p* < .001, for Source Leaning *r*(722) = .58, *p* < .001, for Expertise *r*(722) = .38, *p* < .001, and for the crime policy Hypothesis, *r*(722) = .51, *p* < .001. The Brier scores were .07 for Bias Intensity, .06 for Source Leaning, .05 for Expertise, and .04 for the crime policy Hypothesis. VMs were 27% for Bias Intensity, 94% for Source Leaning, 19% for Expertise, and 88% for the crime policy Hypothesis.

### 3.4 Trends analysis

Like in Study 1, we conducted analyses of the predicted and observed trends caused by our manipulations. These analyses were conducted *post hoc*, and were not preregistered. To perform the analyses, we constructed an independent variable called “Ev_Support,” short for “Evidentiary Support,” which refers to how well‐supported the source's first claim was by the evidence presented for it. This variable increases linearly from 1 to 5 as the degree of support increases. When the claim was conflicting with the evidence (i.e., the claim was that the policy was “harmful” but the evidence said the policy was beneficial, or vice versa), Ev_Support was 1 if the evidence was strong, and 2 if the evidence was weak. When the evidence was neutral, Ev_Support was 3. When the claim was consistent with the evidence (both agreeing the policy was harmful, or both agreeing it was beneficial), Ev_Support was 4 if the evidence was weak, and 5 if the evidence was strong.

**Fig. 8:**
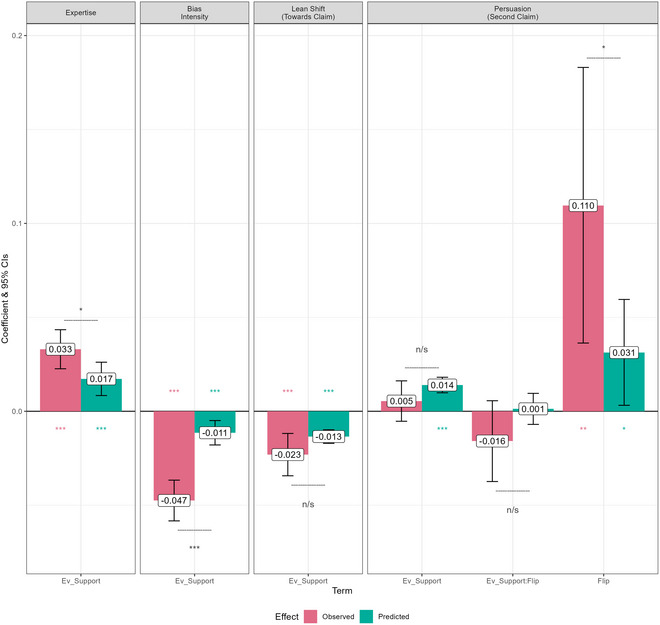
Predicted and observed trends in Study 2. Colored stars indicate the significance of the corresponding observed or predicted effect's coefficient, black stars indicate the significance of the Ev_Support:Type interaction. *Note*. *** = *p* < .001; ** = *p* < .010; * = *p* < .05; n/s = *p* > .05.

For Expertise and Bias Intensity, we found the predicted and observed trends by performing separate multi‐level regressions for the predicted and observed amounts of updating (posterior − prior) for each characteristic. We used Ev_Support as a fixed‐effects predictor, and included a random intercept for the source's claim. Since higher Ev_Support should push the participant's prior further in the direction of the source's claim before they receive it, it should lead them to attribute lower Bias Intensity and higher Expertise to the source, as Figs. 2B and D show, respectively (and as the trends in Fig. 8A indicate). To statistically compare the trends, we then pooled, separately for each characteristic, the predicted and observed updating scores into one dataset and performed another multi‐level regression, introducing a fixed‐effects interaction between Ev_Support and Type (predicted vs. observed) in addition to the terms in the individual regressions.

For Source Leaning, what should happen is a little more complicated, as the direction of updating depends on the claim. Whatever claim the source makes, the participant should update their perception of the source's Leaning further in the direction consistent with that claim (pro‐Government if they say the policy is beneficial, anti‐Government if they say it is harmful), but the *magnitude* of this shift should also depend on Ev_Support, as greater Ev_Support reduces the participant's need to explain away the source's claim by positing bias. Therefore, we construct a measure called “Lean Shift” which measures the difference between posterior and prior estimates of Source Leaning in the expected direction: posterior − prior when the testimony is “beneficial,” and prior − posterior when the testimony is “harmful.” With Lean Shift as the DV, we perform the same set of multi‐level regressions as for Expertise and Bias Intensity.

For Hypothesis updating in response to the second claim, similar transformations were necessary, as obviously, the direction of updating depends upon the claim. We, therefore, calculated a “Persuasion” measure and used it as the dependent variable, where when the second claim was “beneficial,” Persuasion = posterior − prior, and when the second claim was “harmful,” Persuasion = prior − posterior. We performed similar but slightly different multi‐level regressions than for the previous three variables, as we interacted Ev_Support with a variable called “Flip,” which coded for whether the direction of the participant's testimony “flipped” from the first claim to the second claim—if it did (e.g., first claim = “beneficial,” second claim = “harmful,” or vice versa), Flip was coded 0.5, whereas if both claims were the same, Flip was coded −0.5. This “sum contrast” coding scheme allows the main effect of Ev_Support to be calculated as the average of the effects across both levels of Flip, rather than treating one level as the baseline, providing comparability to the regressions for the previous three variables. We included random intercepts for both the first and second claims.

Fig. 8 shows the results of these trends, reporting the Observed (green) and Predicted (red) coefficient for Ev_Support for each variable (and coefficients for the additional fixed terms for Persuasion), as well as showing whether the Ev_Support:Type interaction was significant using significance stars (in black). As it shows, for five out of the six coefficients, the predictions and observations agree in terms of the existence and direction of the trends, though in three of these cases, the observed effect is stronger. The exceptional case is for the effect of Ev_Support on Persuasion, where an effect which is predicted to be statistically significant is in fact nonsignificant, though this discrepancy is mollified by the fact that the predicted and observed trends do not differ from each other.

### 3.5 Discussion

We obtained results consistent with all four preregistered hypotheses—the model's predictions correlate significantly with the observations for every variable. However, *post hoc* analyses showed there were some statistically significant discrepancies between the predicted and observed effects of our manipulations. The effect of the degree of support for the source's first claim (Ev_Support) on how much participants updated their perceptions of the source's Expertise and Bias Intensity was stronger than predicted by the model, and the effect of a person “flipping” the direction of their testimony between claims on the persuasiveness of their second claim was stronger than expected too. The effect of Ev_Support on the persuasiveness of the second claim (about the crime policy) was null rather than positive as predicted, though the two trends did not actually differ with statistical significance, making this discrepancy less problematic. Overall, it seems that the model is better at predicting trends qualitatively than quantitatively—though equally, where there are significant differences in the trends, the quantitative differences remain small, given that estimates were made on a 100‐point scale. For Expertise, the difference is only 1.6 points for each unit of Ev_Support (*b* = 0.033 vs. 0.017), and for Bias Intensity, only 3.6 points per unit (*b* = −0.047 vs. −0.011), whereas the effect of the binary Flip variable is 7.9 points stronger than expected (*b* = 0.110 vs. 0.031).

Study 2 also contains more evidence of belief updating patterns that are not consistent with the Reliability and Trustworthiness‐and‐Expertise models. The fact that the Flip variable strongly increases persuasion cannot be accommodated by these models, as they cannot encode any information about the direction that a source's claim is expected to take; therefore, the fact that a source's claim is surprising given their leaning cannot be encoded in the model. But applying the logic of the Bias‐and‐Expertise model, making two claims with inconsistent directions means the second claim goes against the source's presumed bias direction, as implied by the first claim, suggesting the hypothesis is so likely to be true it has overpowered the source's bias (see Fig. 2A).

## 4 General discussion

We developed a Bayesian Network model of source effects, the Bias‐and‐Expertise model, which integrates the critical source characteristic for political information sources, bias. The model demonstrates predictive validity across two studies, for predictions of four of its highest‐level variables: Hypothesis, Expertise, Source Leaning, and Bias Intensity. We consistently observed statistically significant correlations between observed and predicted posterior judgments, and saw that the model was good at qualitatively predicting the trends in updating that our manipulations caused, though sometimes observed effects were smaller or larger than predicted. Absolute fits between observed and predicted posteriors were also, while not perfect, relatively accurate, particularly given that the model contained no free parameters, and participants made judgments on a 100‐point scale.

These results demonstrate that the Trustworthiness‐and‐Expertise model (Hahn et al., 2013; Harris et al., 2016; Madsen et al., 2018) and Reliability model (Collins et al., 2018; Merdes et al., 2021; Olsson, 2011, 2013), while no doubt applicable in contexts where reliability, expertise, and trustworthiness are the central constructs of source credibility, are less applicable than the Bias‐and‐Expertise Model to politics. Both studies demonstrate effects our model can predict, but these models cannot, where sources who make claims which conflict with their leaning are more persuasive, in line with empirical findings elsewhere (Eagly et al., 1978; Wallace et al., 2020a; Walster et al., 1966).

Future studies could apply our model to the study of polarization. A classic idea in political psychology is that partisans filter information about politics through a “perceptual screen,” an interpretative bias in favor of their “partisan orientation” (Campbell, Converse, Miller, & Stokes, 1980 [1960], p. 133). Indeed, Republicans and Democrats display many “partisan gaps,” holding opposing beliefs on a range of issues which are congenial to their party's agenda—for example, 50% more Republicans than Democrats say that illegal immigrants have a higher crime rate than nonimmigrants (Peterson & Iyengar, 2021), and when the President belongs to the opposing party, US partisans rate the economy as performing worse (Bartels, 2002). The dominant position is that partisan's interpretive biases emerge from identity‐driven motivated reasoning, with partisans finding ways to distort the available evidence so as to satisfy their desire to hold beliefs which are congenial to “their” party (Bolsen, Druckman, & Cook, 2014; Campbell et al., 1980; Kahan, Hoffman, Braman, Evans, & Rachlinski, 2012; Van Bavel, Rathje, Vlasceanu, & Pretus, 2024; Williams, 2021). Yet, bias perceptions could create a purely cognitive “perceptual screen.” If partisans explain their opponents’ tendency to make claims about politics and morality that are, in their view, *wrong*, by attributing biases to them in the form of, for example, consumption of biased media and having incorrect ways of thinking, all subsequent claims they hear their opponents make are filtered through a prism of presumed bias and are, therefore, likely to be dismissed as irrelevant to the truth. Therefore, when exposed to conflicting claims from their opponents and their allies—who they think are less biased simply because what they say usually appears, in their view, *correct*—people will update more in the direction of their allies’ claims. Over time, this will cause people with beliefs on opposing sides of contested issues to grow further apart in their beliefs and bias perceptions. This proposal is similar to a cyclical model of rational belief polarization recently proposed by Young et al. (2025), which focuses on perceptions of testimonial dependence as the key mediator; since perceptions of bias pertain to individuals rather than groups, and are somewhat conceptually simpler, the perception of bias may be a stronger and more prevalent mediator.

Indeed, Schwalbe et al. (2020) provide hints that bias perceptions may drive polarization in the real world—they found that over the course of several weeks in the 2016 US Presidential campaign, how people's support for the candidates shifted was predicted by their initial perceptions of the “objectivity” of Democrats and Republicans, though Schwalbe et al. (2020) did not propose a formal cognitive model for why this might occur. It would, therefore, be useful to determine the extent to which the model of polarization sketched above is plausible.

While some previous authors have attempted to contrast Bayesian and motivated cognition models in the study of partisan gaps (Bartels, 2002; Bullock, 2009; Gerber & Green, 1999), none have considered the role of bias perceptions. Critically, Bartels’ (2002) supposition that if partisans were Bayesian, their political beliefs would converge over time is not necessarily correct if partisans have differential bias perceptions. As identity‐motivated biases have also been linked to susceptibility to political misinformation (Martel et al., 2024), determining whether bias perceptions play a role in people's tendency to have greater trust in fake news headlines that come from co‐partisan sources (Traberg & van der Linden, 2022) could be an avenue for future research.

The Bias‐and‐Expertise Model could also help explain the party elite cue effect (AKA the “party over policy effect”) (Cohen, 2003; Tappin, Berinsky, & Rand, 2023), wherein partisans tend to shift their stated policy positions toward the positions they are told their party's elites occupy. While this is often attributed to an identity‐driven bias that causes people to *want* to agree with their party (e.g., Kahan, 2016), it could simply reflect that people trust their party elites’ ability to adopt good positions on policy issues in a relatively unbiased manner. Tappin et al. (2023) find that when US partisans are told the positions of *both* Trump and Biden, which are conflicting, they tend to shift their own positions toward the leader of the party which they most closely identify with—this polarization could be because partisans, as the model predicts, are likely to attribute much less bias to the leader of their own party due to their experience of agreeing with that leader more on previous issues, and should, therefore, update more strongly to their cue. Future studies could investigate whether bias perceptions mediate the party elite cue effect.

But while the Bias‐and‐Expertise Model has been developed and tested with political contexts primarily in mind, it can be applied to testimony provided by any kind of potentially biased source, and so could be used for research in other domains where biased sources may be present, like financial negotiations, legal cases, advertising, and science communication.

The model and the evidence for its predictive validity are not without limitations, however. While the correlations between predictions and observations for Hypotheses (.61 in Study 1, .51 in Study 2) and Source Leaning were large (.58), those for Bias Intensity and Expertise were weaker (.16 and .38, respectively). Notably, the Supplementary Study also finds that posterior judgments for all three source characteristics correlated significantly with model‐based predictions, but that the correlations for Bias Intensity and Expertise were lower than Source Leaning, and highly sensitive to a methodological choice about how to estimate the priors. Predictions of Bias Intensity and Expertise may be noisier than others because they are less intuitive constructs—bias is usually associated with a particular direction, so asking for a general perception of whether a source is biased *at all* may present a difficult task that is error‐prone. Similarly, our measure of bias does not distinguish between intentional and nonintentional biases—again, people may find it difficult to collapse perceptions of these two constructs into one. This possibility seems even more pertinent with Expertise, where participants need to estimate the counterfactual of how accurate the source would be if they were not biased. It may be beneficial for future studies, therefore, to test different ways of measuring perceived Bias Intensity and Expertise to ascertain the method that returns the strongest correlations between predictions and observations. This method could then be used to measure any priors for use in simulations and predictions of effect size. Or, an alternative paradigm entirely could be used: elsewhere in psychology, Bayesian cognitive models are tested using paradigms that do not require participants to provide explicit judgments of probabilities; rather, their priors and updating are inferred from observing their behavior across many trials (e.g., Lawson, Mathys, & Rees, 2017). If such an approach could be applied to source effects models, perhaps using a design where participants had to make decisions based on source testimony, it would also avoid the problem that arises from asking participants to introspect upon their confidence and translate it into a 0−100 scale, which is likely to be noisy. This approach could then be used to adjust the model until it is maximally predictive, making it ideal for simulation‐based work.

The current evidence would be strengthened if it were replicated using first‐person paradigms and in real‐world political scenarios. We avoided using real‐world scenarios in this first study of the model's validity, as if participants were asked to make judgments about real‐world sources and issues, and it turned out the model performed poorly, it would be ambiguous as to whether this is because to the extent that sources reason about political information sources rationally, the Bias‐and‐Expertise Model fails to capture that rationality, or because their reasoning is not rational, but rather is compromised, as widely theorized, by identity‐driven biases (e.g., van Bavel & Pereira, 2018). Having now established that the model performs sufficiently well for artificial scenarios, future studies can test how well the model performs using real‐world stimuli. It would also be helpful for assessing the performance of the model if there were greater consensus in the literature about methods for evaluating the predictive validity of Bayesian Networks, including both the ideal tests, and benchmarks to differentiate between differing levels of prediction quality. There are also behaviors of the model that have not been tested, like predicting responses to sources’ claims about neutral stimuli. Further progress in these areas would help to validate the model.

We also note a few discrepancies between our predictions and observations, which future studies could probe further to ascertain whether they represent random deviations, or systematic deviations that would require some adjustment to the model. The first is that in Study 1, we observed that for Low Expertise sources, those who were Impartial were slightly less persuasive than sources who made a claim that conflicted with their leaning, though the model numerically predicted they should be slightly more persuasive. Given that the model did not predict differences here that were statistically significant, we do not regard this as a major discrepancy. Neither kind of source should *always* be more persuasive than the other—it depends on the participants’ priors for their respective Expertise and the biased source's Bias Intensity—so discrepancies could arise from inaccurately reported priors or from an error in the model. Future studies could investigate whether this discrepancy is robust and try to tease apart the different possibilities for why it occurs, if so. In Study 2, there were a few discrepancies, including that posterior Bias Intensity judgments were more extreme than expected for sources for whose claim there was neutral evidence, even relative to the regression line fitted to the observed posterior Bias Intensity judgments. Again, whether this effect is robust and what causes it could be addressed by future studies. Until such time as this research can be performed, the discrepancies appear minor enough for the model to be retained in its current form, albeit with an awareness of these discrepancies.

To conclude, the two studies discussed in this paper demonstrate that the Bias‐and‐Expertise model has adequate predictive validity, though ideally its noisiness would be reduced, and that it can account for numerous known patterns of belief updating related to source bias. The model offers an advance on existing Bayesian Network models of source effects for application to politics, as it can explain a phenomenon for which we obtained robust evidence—that biased sources are more persuasive when the claims they make conflict with the direction of their presumed bias—which existing models cannot.

## Supporting information



Supporting Information

## Notes

1 Eagly, Wood, and Chaiken (1978) also treat learning from a source as a causal inference problem, but do not take a Bayesian approach.

2 For this reason, we model the nodes in our network as binary variables, that is, variables which have two possible states which they occupy probabilistically, and model the beliefs as point estimates, rather than modeling the nodes as continuous variables with (e.g.) beta distributions for beliefs.

3 “Logit” just means converting a probability, *p*, into a ratio of the form *p*/(1−*p*), then taking the log of that ratio.

4 We were also interested in the impact on the predictiveness of the model that would come from using participants’ *estimates* of the conditional probabilities in Table 2 rather than assuming them, which neither Madsen (2019) or Harris, Hahn, Madsen, and Hsu (2016) had analyzed. Analyses using these estimated probabilities for Study 1 are discussed in Supplementary Material 1, showing that the model's predictiveness is somewhat reduced, presumably because it introduces substantial additional noise into the predictions. This was not repeated for Study 2.

5 In Qualtrics, everything on the same page is visible at the same time, though participants may need to scroll down to see it; participants have to click forward to navigate to the next page and cannot go back to previous pages.

6 In the preregistration, we refer to measuring the source's Bias Direction instead of Source Leaning. Because the Hypothesis Leaning is always fixed at 1, Bias Direction and Source Leaning are necessarily identical, therefore, predictions for one equally pertain to the other. We have switched to referring to Source Leaning in this article, however, as a purely semantic choice, because we asked participants to estimate how much sources were pro‐ or anti‐Government, which is conceptually a Source Leaning judgment rather than a Bias Direction judgment (which just measures whether they are “For” or “Against” the hypothesis).

7 We actually preregistered to test the correlation for Bias Direction rather than Source Leaning, but the predicted correlation is necessarily identical for both variables—see Footnote 6.
