# Supplementary material for: The Bias‐and‐Expertise Model: A Bayesian Network Model of Political Source Characteristics
Source: Cogn Sci. 2025 Nov 19;49(11):e70141. doi: 10.1111/cogs.70141 (PMC12631054; doi:10.1111/cogs.70141)
Supplement: Supplementary file 1 — Supporting Information [file COGS-49-e70141-s001.docx]

**The Bias-and-Expertise Model: A Bayesian Network Model of Political Source Characteristics – Supplementary Materials**

**Contents**

Supplementary Material 1: Results using estimated conditionals probabilities Pages 2-5

Supplementary Material 2: Disaggregated Analysis of Study 1 Pages 6-8

Supplementary Material 3: Supplementary Study Pages 9-18

Supplementary Material 4: Latin Square for Study 1 Randomisation Page 19

**Supplementary Material 1: Results using estimated conditionals probabilities**

***Questions used to elicit conditional probability estimates***

Different question wordings were used to elicit participants’ conditional probability estimates in each study. These are shown in Tables S1-S3.

**Table S1**

*Questions used in Study 1 – Version A*

| **Hyp.** | **Exp.** | **Bias** | **Wording** |
| --- | --- | --- | --- |
| Beneficial | High | For | If an expert who was biased towards the Government was asked whether the Government's new policy would be beneficial or harmful to the country, and in reality it was beneficial, what is the chance that they would, if asked, say it was beneficial? |
|  |  | Impartial | If an impartial expert was asked whether the Government's new policy would be beneficial or harmful to the country, and in reality it was beneficial, what is the chance that they would, if asked, say it was beneficial? |
|  |  | Against | If an expert who was biased against the Government was asked whether the Government's new policy would be beneficial or harmful to the country, and in reality it was beneficial, what is the chance that they would, if asked, say it was beneficial? |
|  | Low | For | If a person who had no expertise about government policy and was biased towards the government was asked whether the Government's new policy would be beneficial or harmful to the country, and in reality it was beneficial, what is the chance that they would, if asked, say it was beneficial? |
|  |  | Impartial | If an impartial person who had no expertise about government policy was asked whether the Government's new policy would be beneficial or harmful to the country, and in reality it was beneficial, what is the chance that they would, if asked, say it was beneficial? |
|  |  | Against | If a person who had no expertise about government policy and was biased against the government was asked whether the Government's new policy would be beneficial or harmful to the country, and in reality it was beneficial, what is the chance that they would, if asked, say it was beneficial? |
| Harmful | High | For | If an expert who was biased towards the Government was asked whether the Government's new policy would be beneficial or harmful to the country, and in reality it was harmful, what is the chance that they would, if asked, say it was harmful? |
|  |  | Impartial | If an impartial expert was asked whether the Government's new policy would be beneficial or harmful to the country, and in reality it was harmful, what is the chance that they would, if asked, say it was harmful? |
|  |  | Against | If an expert who was biased against the Government was asked whether the Government's new policy would be beneficial or harmful to the country, and in reality it was harmful, what is the chance that they would, if asked, say it was harmful? |
|  | Low | For | If a person who had no expertise about government policy and was biased towards the government was asked whether the Government's new policy would be beneficial or harmful to the country, and in reality it was harmful, what is the chance that they would, if asked, say it was harmful? |
|  |  | Impartial | If an impartial person who had no expertise about government policy was asked whether the Government's new policy would be beneficial or harmful to the country, and in reality it was harmful, what is the chance that they would, if asked, say it was harmful? |
|  |  | Against | If a person who had no expertise about government policy and was biased against the government was asked whether the Government's new policy would be beneficial or harmful to the country, and in reality it was harmful, what is the chance that they would, if asked, say it was harmful? |

**Table S2**

*Questions used in Study 1 – Version B*

| **Hyp.** | **Exp.** | **Bias** | **Wording** |
| --- | --- | --- | --- |
| Beneficial | High | For | Consider the following a kind of person: If they could avoid being biased, this person would be an expert on policy issues If this person was biased, they would be biased towards the government party This person is biased when discussing policy issues Imagine they are asked whether the Government's new policy will be beneficial or harmful. What is the chance they would say a beneficial policy will be beneficial? |
|  |  | Impartial | Consider the following a kind of person: If they could avoid being biased, this person would be an expert on policy issues. This person is impartial when discussing policy issues. Imagine they are asked whether the Government's new policy will be beneficial or harmful. What is the chance they would say a beneficial policy will be beneficial? |
|  |  | Against | Consider the following a kind of person: If they could avoid being biased, this person would be an expert on policy issues If this person was biased, they would be biased towards the opposition party This person is biased when discussing policy issues Imagine they are asked whether the Government's new policy will be beneficial or harmful. What is the chance they would say a beneficial policy will be beneficial? |
|  | Low | For | Consider the following a kind of person: Even if they could avoid being biased, this person would still not be an expert on policy issues If this person was biased, they would be biased towards the government party This person is biased when discussing policy issues Imagine they are asked whether the Government's new policy will be beneficial or harmful. What is the chance they would say a beneficial policy will be beneficial? |
|  |  | Impartial | Consider the following a kind of person: Even if they could avoid being biased, this person would still not be an expert on policy issues. This person is impartial when discussing policy issues. Imagine they are asked whether the Government's new policy will be beneficial or harmful. What is the chance they would say a beneficial policy will be beneficial? |
|  |  | Against | Consider the following a kind of person: If they could avoid being biased, this person would be an expert on policy issues If this person was biased, they would be biased towards the opposition party This person is biased when discussing policy issues Imagine they are asked whether the Government's new policy will be beneficial or harmful. What is the chance they would say a beneficial policy will be beneficial? |
| Harmful | High | For | Consider the following a kind of person: If they could avoid being biased, this person would be an expert on policy issues If this person was biased, they would be biased towards the government party This person is biased when discussing policy issues Imagine they are asked whether the Government's new policy will be beneficial or harmful. What is the chance they would say a harmful policy will be harmful? |
|  |  | Impartial | Consider the following a kind of person: If they could avoid being biased, this person would be an expert on policy issues. This person is impartial when discussing policy issues. Imagine they are asked whether the Government's new policy will be beneficial or harmful. What is the chance they would say a harmful policy will be harmful? |
|  |  | Against | Consider the following a kind of person: Even if they could avoid being biased, this person would still not be an expert on policy issues If this person was biased, they would be biased towards the opposition party This person is biased when discussing policy issues Imagine they are asked whether the Government's new policy will be beneficial or harmful. What is the chance they would say a harmful policy will be harmful? |
|  | Low | For | Consider the following a kind of person: Even if they could avoid being biased, this person would still not be an expert on policy issues If this person was biased, they would be biased towards the government party This person is biased when discussing policy issues Imagine they are asked whether the Government's new policy will be beneficial or harmful. What is the chance they would say a harmful policy will be harmful? |
|  |  | Impartial | Consider the following a kind of person: Even if they could avoid being biased, this person would still not be an expert on policy issues. This person is impartial when discussing policy issues. Imagine they are asked whether the Government's new policy will be beneficial or harmful. What is the chance they would say a harmful policy will be harmful? |
|  |  | Against | Consider the following a kind of person: Even if they could avoid being biased, this person would still not be an expert on policy issues If this person was biased, they would be biased towards the opposition party This person is biased when discussing policy issues Imagine they are asked whether the Government's new policy will be beneficial or harmful. What is the chance they would say a harmful policy will be harmful? |

**Table S3**

*Questions used in Supplementary Study*

| **Hyp.** | **Exp.** | **Bias** | **Wording** |
| --- | --- | --- | --- |
| Beneficial | High | For | If a media organisation that was biased towards the Government, but, when they could avoid being biased, was very knowledgeable about policy issues was asked whether the Government's new policy would be beneficial or harmful to the country, and in reality it was beneficial, what is the chance that they would say it was beneficial? |
|  |  | Impartial | If an impartial media organisation that was very knowledgeable about policy issues was asked whether the Government's new policy would be beneficial or harmful to the country, and in reality it was beneficial, what is the chance that they would say it was beneficial? |
|  |  | Against | If a media organisation that was biased against the Government, but, when they could avoid being biased, was very knowledgeable about policy issues was asked whether the Government's new policy would be beneficial or harmful to the country, and in reality it was beneficial, what is the chance that they would say it was beneficial? |
|  | Low | For | If a media organisation that was biased towards the Government, and, even when they could avoid being biased, was not at all knowledgeable about policy issues was asked whether the Government's new policy would be beneficial or harmful to the country, and in reality it was beneficial, what is the chance that they would say it was beneficial? |
|  |  | Impartial | If an impartial media organisation that was not at all knowledgeable about policy issues was asked whether the Government's new policy would be beneficial or harmful to the country, and in reality it was beneficial, what is the chance that they would say it was beneficial? |
|  |  | Against | If a media organisation that was biased against the Government, and, even when they could avoid being biased, was not at all knowledgeable about policy issues was asked whether the Government's new policy would be beneficial or harmful to the country, and in reality it was beneficial, what is the chance that they would say it was beneficial? |
| Harmful | High | For | If a media organisation that was biased towards the Government, but, when they could avoid being biased, was very knowledgeable about policy issues was asked whether the Government's new policy would be beneficial or harmful to the country, and in reality it was harmful, what is the chance that they would say it was harmful? |
|  |  | Impartial | If an impartial media organisation that was very knowledgeable about policy issues was asked whether the Government's new policy would be beneficial or harmful to the country, and in reality it was beneficial, what is the chance that they would say it was beneficial? |
|  |  | Against | If a media organisation that was biased against the Government, but, when they could avoid being biased, was very knowledgeable about policy issues was asked whether the Government's new policy would be beneficial or harmful to the country, and in reality it was harmful, what is the chance that they would say it was harmful? |
|  | Low | For | If a media organisation that was biased towards the Government, and, even when they could avoid being biased, was not at all knowledgeable about policy issues was asked whether the Government's new policy would be beneficial or harmful to the country, and in reality it was harmful, what is the chance that they would say it was harmful? |
|  |  | Impartial | If an impartial media organisation that was not at all knowledgeable about policy issues was asked whether the Government's new policy would be beneficial or harmful to the country, and in reality it was harmful, what is the chance that they would say it was harmful? |
|  |  | Against | If a media organisation that was biased against the Government, and, even when they could avoid being biased, was not at all knowledgeable about policy issues was asked whether the Government's new policy would be beneficial or harmful to the country, and in reality it was harmful, what is the chance that they would say it was harmful? |

To input these elicited probability estimates into the model, the hypothesis was modelled as “The policy is beneficial”. Therefore testimony that the policy was beneficial was modelled as “True”, and testimony that the policy was harmful was modelled as “False”. Therefore, the elicited probabilities were inputted into the model as follows:

**Table S4**

*How the elicited conditional probability estimates are used in the model.*

| **Hyp.** | **Exp.** | **Bias** | **Conditional Probability** |
| --- | --- | --- | --- |
| Beneficial | High | For | *p*(Testimony = TRUE\|Exp. = 1, Hyp. = True, Bias Int. = 1, Bias Dir. = For) |
|  |  | Impartial | *p*(Testimony = TRUE\|Exp. = 1, Hyp. = True, Bias Int. = 0) |
|  |  | Against | *p*(Testimony = TRUE\|Exp. = 1, Hyp. = True, Bias Int. = 1, Bias Dir. = Against) |
|  | Low | For | *p*(Testimony = TRUE\|Exp. = 0, Hyp. = True, Bias Int. = 1, Bias Dir. = For) |
|  |  | Impartial | *p*(Testimony = TRUE\|Exp. = 0, Hyp. = True, Bias Int. = 0) |
|  |  | Against | *p*(Testimony = TRUE\|Exp. = 0, Hyp. = True, Bias Int. = 1, Bias Dir. = Against) |
| Harmful | High | For | *p*(Testimony = FALSE\|Exp. = 1, Hyp. = False, Bias Int. = 1, Bias Dir. = For) |
|  |  | Impartial | *p*(Testimony = FALSE\|Exp. = 1, Hyp. = False, Bias Int. = 0) |
|  |  | Against | *p*(Testimony = FALSE \|Exp. = 1, Hyp. = False, Bias Int. = 1, Bias Dir. = Against) |
|  | Low | For | *p*(Testimony = FALSE \|Exp. = 0, Hyp. = False, Bias Int. = 1, Bias Dir. = For) |
|  |  | Impartial | *p*(Testimony = FALSE \|Exp. = 0, Hyp. = False, Bias Int. = 0) |
|  |  | Against | *p*(Testimony = FALSE \|Exp. = 0, Hyp. = False, Bias Int. = 1, Bias Dir. = Against) |

To calculate the conditional probability of a source saying that the policy is harmful when in fact it is beneficial for a given permutation of the other variables, the corresponding probability in the table of the source saying the policy is beneficial can be subtracted from 1. Similarly, to calculate the conditional probability of a source saying that the policy is beneficial when in fact it is harmful for a given permutation of the other variables, the corresponding probability in the table of the source saying the policy is harmful can be subtracted from 1.

***Results using individual conditional probability estimates***

Each participant’s posterior in each condition was calculated using their specific estimates of the conditional probabilities. Overall model performances results using these predicted estimates are shown in Table S5. Note that in Study 1, three participants didn’t estimate some of the conditionals, reducing the sample size for these analyses.

**Table S5**

*Results of the main article’s primary analyses when predictions are derived using each participant’s own estimates of the conditional probabilities in the network from the main article’s Table 2.*

| **Study** | **Variable** | ***r*** | **Mean individual *r*** | **Brier** | **VM** |
| --- | --- | --- | --- | --- | --- |
| 1 | Hypothesis | 0.46, *p* < .001 | 0.48 | 0.046 | 91% |
| 1 version A | Hypothesis | 0.52, *p* < .001 | 0.59 | 0.045 | 86% |
| 1 version B | Hypothesis | 0.46, *p* < .001 | 0.36 | 0.047 | 92% |
| Supplementary | Bias Intensity (without follow-up priors) | 0.39, *p* < .001 | 0.32 | 0.057 | 80% |
| Supplementary | Bias Direction (without follow-up priors) | 0.58, *p* < .001 | 0.56 | 0.067 | 96% |
| Supplementary | Expertise (without follow-up priors) | 0.07, *p* = .139 | 0.17 | 0.106 | 50% |
| Supplementary | Bias Intensity (with follow-up priors) | 0.19, *p* < .001 | 0.45 | 0.085 | 79% |
| Supplementary | Bias Direction (with follow-up priors) | 0.52, *p* < .001 | 0.57 | 0.105 | 91% |
| Supplementary | Expertise (with follow-up priors) | 0.53, *p* < .001 | 0.26 | 0.068 | 56% |

**Supplementary Material 2: Disaggregated Analysis of Study 1**

Here we report the main results of Study 1 when the data from Version A and Version B are analyzed separately. Figures S1-S2 show the mean posteriors across conditions, as predicted and observed, with model performance statistics reported below. Figures S3-4 show the predicted and observed trends caused by our manipulations. All methodologies are the same as in the main article, except applied to each version of the study individually.


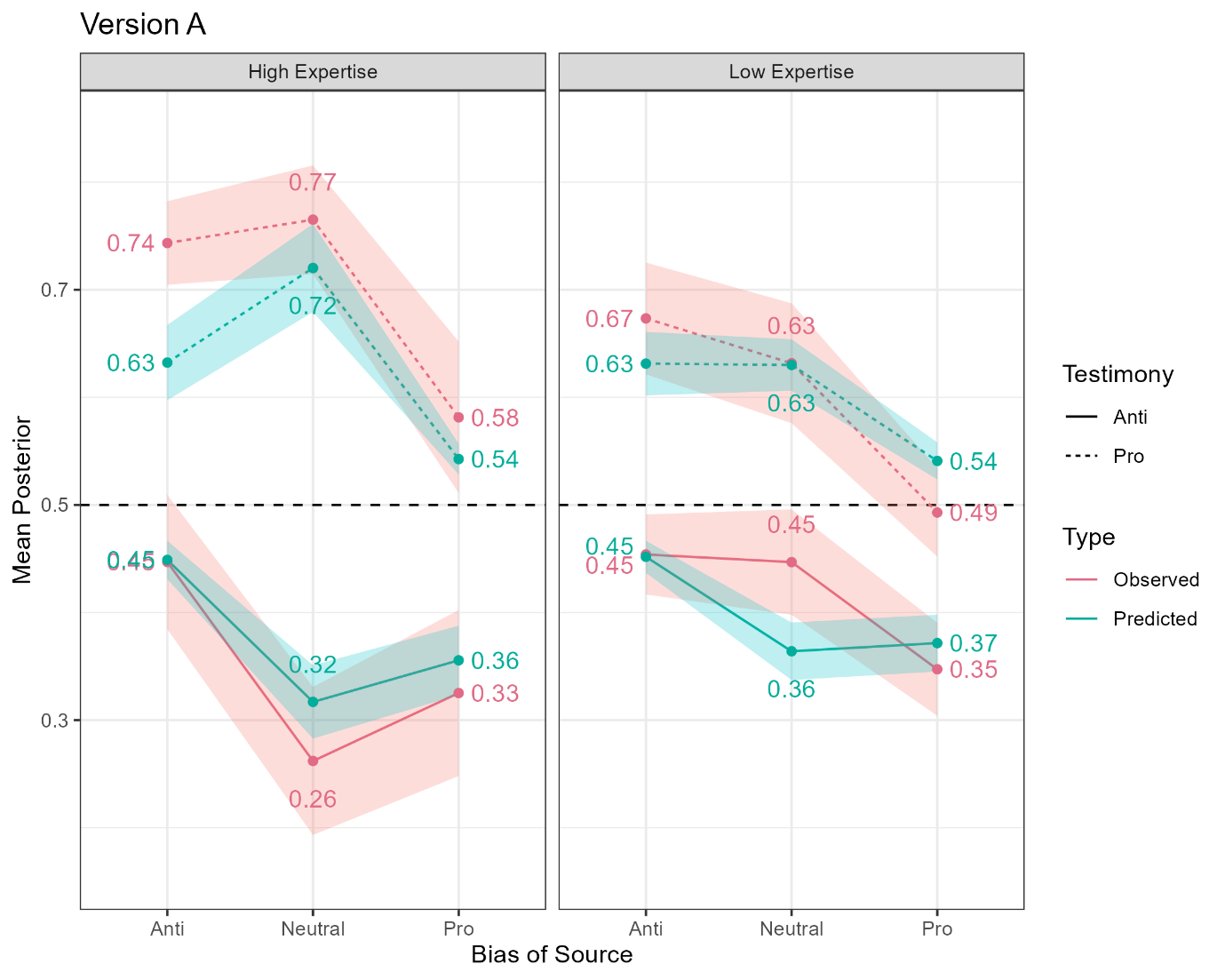


**Figure S1**

*Observed and predicted mean posteriors by condition, Study 1 – Version A.*


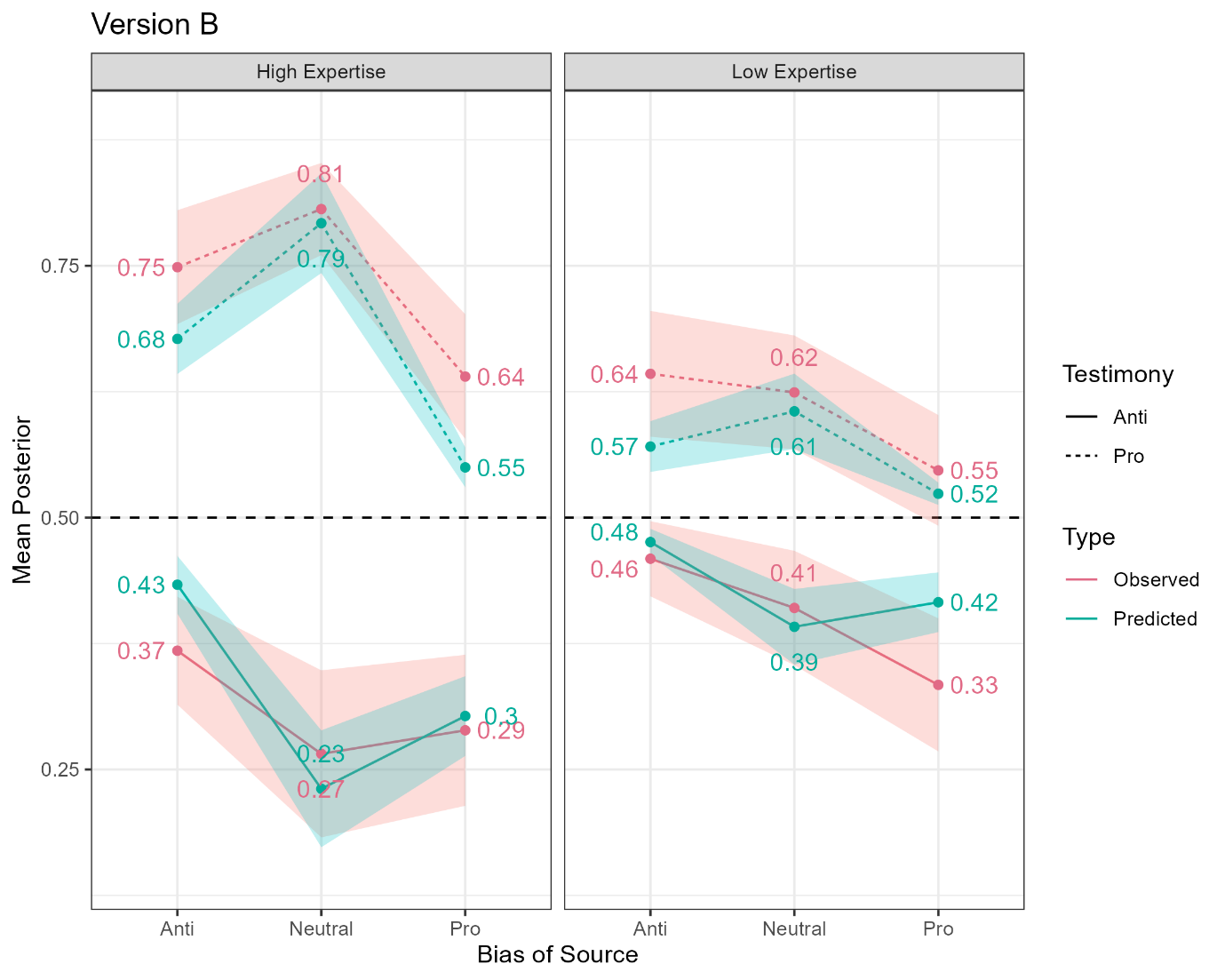


**Figure S2**

*Observed and predicted mean posteriors by condition, Study 1 – Version B.*

**Statistical Measures of Performance:**

***Version A****:*

Correlation: *r*(460) = .610 [.550, .665], *p* < .001.

Mean individual correlation: *r* = .67.

Brier Score (MSE) = .033.

VM = 92.5%

***Version B****:*

Correlation: *r*(406) = .610 [.545, .668], *p* < .001.

Mean individual correlation: *r* = .69.

Brier Score (MSE) = .038.

VM = 92.8%


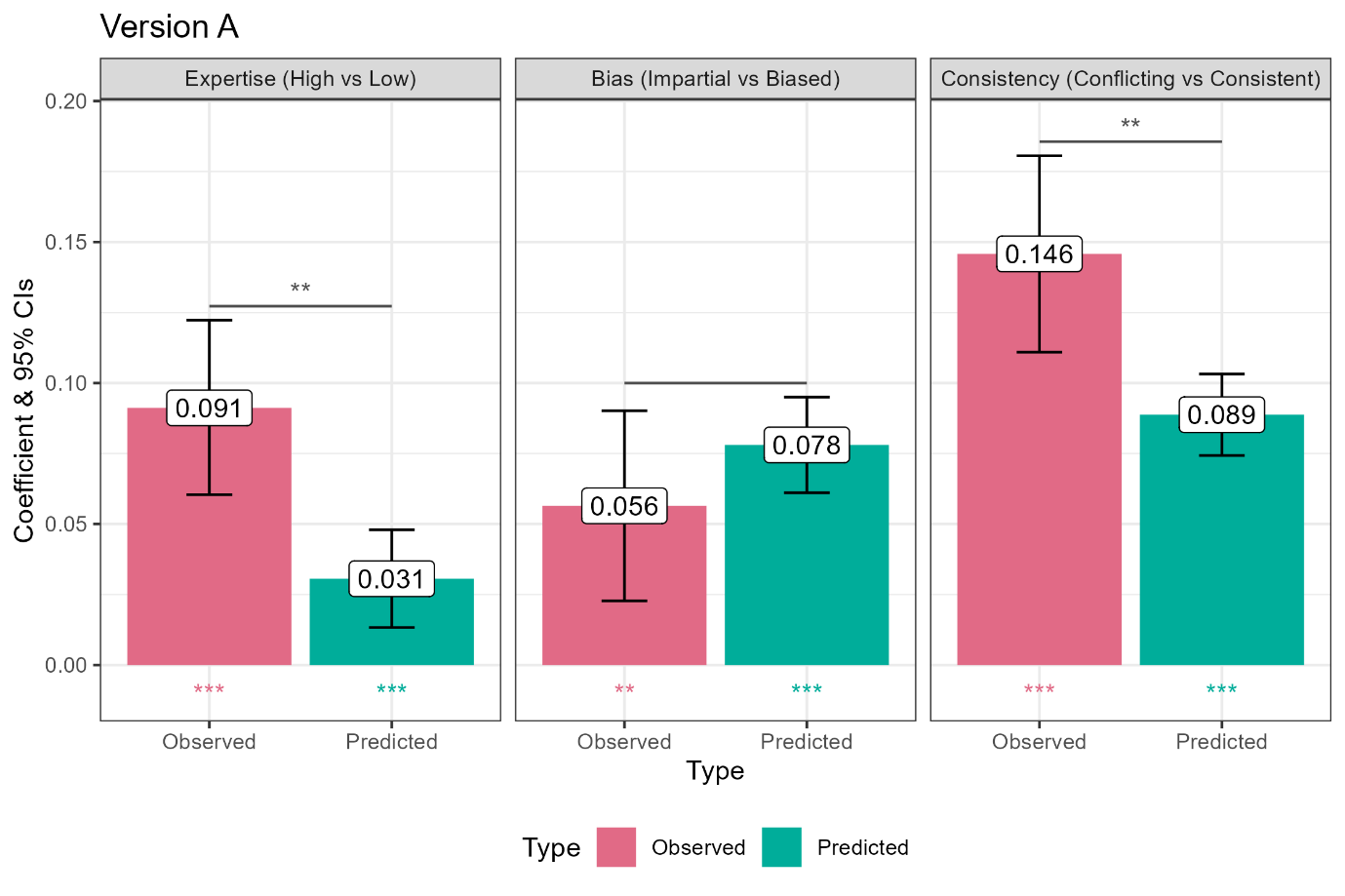


**Figure S3**.

*Trends caused by experimental manipulations – Study 1 Version A.*


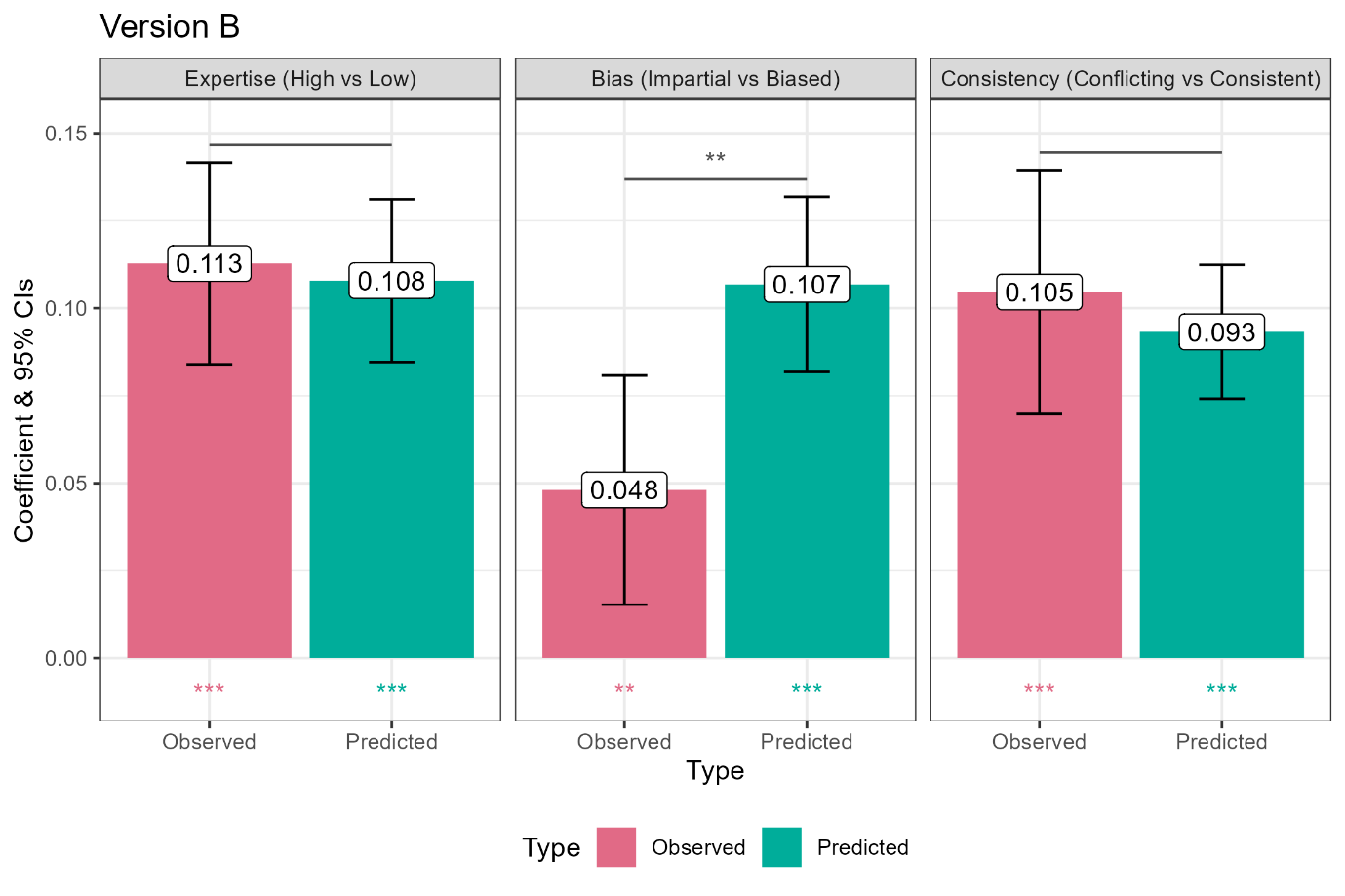


**Figure S4**.

*Trends caused by experimental manipulations – Study 1 Version B.*

**Supplementary Material 3: Supplementary Study**

This study effectively bridges Study 1 and Study 2, exploring belief updating about source perceptions like Study 2, but without any exploration of belief updating regarding hypotheses, while using a design more cosmetically similar to Study 1. We relegate this study to the Supplementary Materials for three reasons. Firstly, there was a programming error which meant that rather than participants completing one trial from each of the 10 experimental conditions in our design space, one trial was included twice and one not at all. Secondly, there is some ambiguity in our results due to us taking two slightly different approaches which seem equally valid but return somewhat different results. This happened because we didn’t measure prior beliefs for the source’s characteristics in our trials, but rather tried to manipulate them such that we could assume what they would be; however, recognizing that our manipulation may not have been successful after collecting the data, we conducted a follow-up study to try to measure what participants’ priors for the source characteristics were, but not all the original participants completed this follow-up. Thirdly, we found evidence that the predictiveness of the model decreased over the course of the experiment, for some dependent variables, which could reflect factors like participant fatigue, inattention, or carry-over effects.

**Method**

***Participants***

We recruited 121 participants, paying £7.50/hour, with a 10p advertised bonus for passing memory checks. We excluded 65 participants in total, leaving a final sample of *N* = 49 (33 failed at least one of six attention checks, which asked for a particular number to be chosen on a slider, 39 failed at least one of four memory checks). Some analyses were performed with data collected in a follow-up study, which 42 of the 49 completed. The total sample consisted of 18 men, 30 women, and one non-binary person. Their median age was 32 with a range of 18-66. We also asked for participants’ ethnicities using the UK census options: 36 selected ‘English, Welsh, Scottish, Northern Irish, or British’, 6 ‘Any other White background’, 2 Bangladeshi, 1 ‘Any other Black, African or Caribbean background’, 1 Chinese, 1 Indian, 1 Irish, and 1 ‘White and Asian’. We informed participants the study had undergone the review procedure of the Department of Psychology Research Ethics Committee at REDACTED FOR BLIND REVIEW before they provided consent.

Again, this study was more than adequately powered. We collected 10 observations per participant, giving 80% power to detect effects as small as *r* = 0.18 (G*Power 3.1.9.4 sensitivity analysis, independent-samples Pearson’s correlation, two-tailed, alpha = 0.05, *n*s = 490; with *ns* = 420 we have 80% power to detect effects as small as *r* = 0.19).

***Design***

As before, James provides Anne with information about whether a new Government policy has been beneficial or harmful to the country. However, he now presents two pieces of information per policy: a piece of evidence about its impact (the conclusion of a team of expert researchers about its effectiveness), and what a media organisation reported about it. Our intention was to employ a 5x2 within-subjects factorial manipulation to vary the strength and direction of the objective evidence (strong harmful, weak harmful, neutral, weak beneficial, strong beneficial) and what the media source reported that the impact of the policy had been (beneficial vs harmful). However, a coding error meant that the trial where the evidence was supposed to be neutral and the source said the policy was beneficial actually presented the evidence as ‘strong beneficial’ – while this trial can just be re-classified to reflect the actual evidence it presented, this error does reduce our coverage of the parameter space somewhat, as we end up with only nine unique kinds of trial.

***Procedure***

After obtaining consent, we told participants, similarly to Study 1, that we would be asking them questions about “political policies in a fictional country”, describing it as “an imaginary Western democracy, which is not the UK or USA”. We said there were only two parties in this country – the Government’s party and Opposition’s party. We warned them there would be attention check questions. Again, we informed them to treat the dialogues separately and not to “use any conclusions you draw from one dialogue to interpret another dialogue”. We also told them “You can assume everything James says is true”.

Then came the main section of the study, in which participants completed 10 experimental trials. Each trial featured a different randomly-selected policy, and a different randomly-selected fictional media organisation. Each trial was split into two phases – a bias induction phase and a testimony phase. The order of events in each trial is depicted in Figure S5. Each trial took place on a different page, and everything pertaining to a single trial was visible at the same time.

Bias Induction Phase

Testimony Phase

**Figure S5**

*Schematic of the events that occurred in each trial in Supplementary Study.*

Before the 10 experimental trials began, we gave participants a dummy trial with a practice memory test after it; participants were told this was just for practice and not excluded for failing it. It involved a trial that looked identical to the experimental trials but was followed by 2 questions, on separate pages, which asked participants to recall what the evidence James presented had been, given a multiple choice of 5 options, and what the media source had said, given a multiple choice of 2 options. Identical memory tests occurred after two of the experimental trials, selected at random – participants were excluded for failing any one of these. Interleaved within the 10 experimental trials was an attention check trial; this was identical to the experimental trials, but every question simply asked participants to give an answer of 20.

After this section, participants completed a block consisting of 12 conditional probability estimates with two attention checks embedded, one asking for a response of 73 and one a response of 12. After this section, participants provided demographic information, were debriefed and thanked, then redirected to Prolific.

***Randomisation***

The order of trials was fully randomised. The choice of policy and media organisation for each trial were randomised using Latin squares.

***Stimuli***

To convey the evidence, James told Anne “I read a report on the policy put together by a team of independent neutral experts and they found there is {…}”, followed by a description of the evidence strength: “strong evidence that it will be beneficial”, “some evidence that it will be beneficial but it’s not conclusive”, “no evidence as to whether it will be harmful or beneficial”, “some evidence that it will be harmful but it’s not conclusive” or “strong evidence that it will be harmful”. These respectively correspond to the strong beneficial, weak beneficial, neutral, weak harmful and strong harmful evidence conditions.

The policies areas were Agricultural, Energy, Education, Crime, Public Security, Anti-corruption, Tax, Housing, Transport, and Foreign Trade. The media organisations were The Daily Booster, The Domestic Chronicle, The Public Globe, The Citizen’s Bugle, Deadline Press, The National Post, The Local Tribune, The Morning Inquirer, DCN News and The Advocate Gazette. We designed the media titles to sound like plausible media organisations while avoiding similarities to any well-known UK news brands.

***Measures***

As before, all measurements were made on 0-100 slider scales which began with a default position of 50. The value which corresponded to the slider’s position was displayed immediately above it.

To measure Anne’s prior belief about the policy’s impact, we asked “In light of the dialogue above, what do you think Anne’s opinion should now be about the government’s **{policy area}** policy?” with the slider labelled “0 = They should be totally confident it’s harmful”, “50 = They should be unsure”, and “100 = They should be totally confident it’s beneficial”.

To measure Bias Intensity, we asked “In light of the dialogue above, what do you think Anne’s opinion should now be about which of the following statements, concerning the media source **{media source}**, is closer to the truth? 100 = “This media source is biased when discussing policy issues” 0 = “This media source is impartial when discussing policy issues””, with the slider labelled “0 = Impartial” and “100 = Biased”.

To measure the source’s Leaning, we asked “And which of the following is closest to the truth? 100 = “If this media source was biased, they would be biased **towards the government**” 0 = “If this media source was biased, they would be biased **against the government**””, with the slider labelled “0 = Against the government” and “100 = Towards the government”. Arguably here we should have been clearer that we wanted participants to estimate Anne’s belief about the source’s leaning, but, as discussed, we expect people to use their own beliefs to determine Anne’s, so it is unlikely to matter.

To measure Expertise, we asked “And which of the following is closest to the truth? 100 = “If they could avoid being biased, this media source **would be an expert** on policy issues” 0 = “Even if they could avoid being biased, this media source **would still not be an expert** on policy issues”, with the slider labelled “0 = Not an expert” and “100 = Expert”. Again, we did not explicitly ask for Anne’s belief about the expertise.

***Follow-Up Study***

After analysing the data that was collected from the study as described above, we realised it might be possible to make more accurate predictions if we knew what people believed Anne’s priors for the sources’ Bias Intensity and Expertise were *before* she heard their testimony. In the study we attempted to force these priors to be 0.5 by having Anne say she had “no opinion” about the media organization, and we assumed they were 0.5 in our predictive modelling. However, if participants themselves had different priors for media organisations’ source bias and expertise, for instance believing media source to generally be biased and lacking expertise, this may have influenced their inferences.

To gather this information, we advertised a follow-up study to the non-excluded participants, 42 of whom participated. They again were paid £7.50 per hour, and provided new informed consent. We gave participants the same task information to recapitulate the experimental scenario, then instructed participants “Imagine a typical media organisation in this country”. We then asked identical Bias Intensity and Expertise questions to those used in the main study for that generic media organisation. We discuss the Results obtained with and without using these priors.

**Results**

We predicted what participants’ source perceptions would be for each trial by inputting their priors for the effectiveness of the policy into the model. For analyses which used the follow-up data, we inputted people’s judgments of the Bias Intensity and Expertise of the typical media source as priors for every source, but assumed the prior for Source Leaning was 0.5. For analyses which did not use the follow-up data, we assumed Bias Intensity, Expertise, and Source Leaning were all 0.5 in every case.


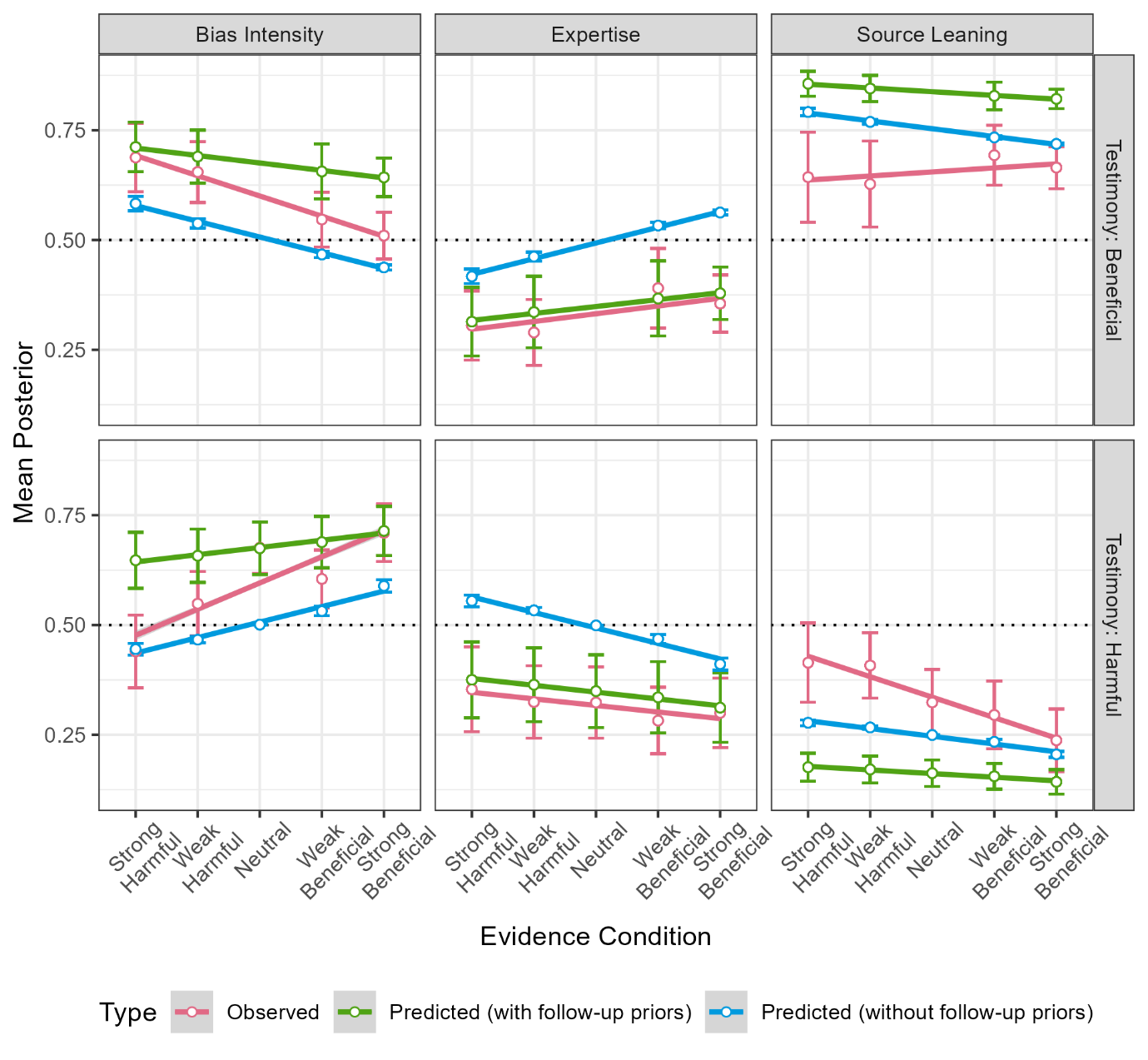


**Figure S6**

*Posterior source perceptions across evidence and testimony conditions – regression lines with means and 95% confidence intervals.*

Figure S6 shows the linear regression lines for the effect of the evidence condition on the posterior source perceptions as observed and as predicted, both using and not using the follow-up data. The figure also shows the mean posterior and 95% CIs. From visual inspection, the predicted trends tend to match those that were observed, though the intercepts clearly differ in several cases.

The Bias Intensity trends observed are similar to those predicted, but with a higher intercept when the follow-up priors are used and a lower one when they are not. The Source Leaning predictions are too extreme in both cases, but more problematic is the fact that the predicted trend appears to go in the opposite direction to the observed trend when the source’s testimony was that the policy had been beneficial. For Expertise, the predicted trend is similar to what was observed, but the intercept is much closer to the observation when the follow-up priors are used; not using the follow-up priors, participants rated the sources as having lower expertise than we had predicted.

**Table S6**

*Performance measures*

|  | Bias Intensity | | Source Leaning | | Expertise | |
| --- | --- | --- | --- | --- | --- | --- |
| Follow-up data used | Y | N | Y | N | Y | N |
| Correlation | 0.19,  *p* < .001 | 0.42,  *p* < .001 | 0.52,  *p* < .001 | 0.58,  *p* < .001 | 0.53,  *p* < .001 | 0.13,  *p* = .005 |
| Brier | .09 | .06 | .10 | .07 | .07 | .10 |
| VM | 80% | 83% | 91% | 95% | 59% | 56% |

Table S6 shows performance measures of our predictions for all three source perceptions, both using the follow-up priors and not. Notably, all correlations between predictions and observations are significant, though sometimes with only small effect sizes (0.1 ≤ *r* < 0.3)^[[1]](#footnote-1)^. It is also evident that whether or not the follow-up priors are used has a strong effect on how well our predictions of Expertise and Bias Intensity correlate with the observations, but, as with the visual inspection, one approach is not consistently better than the other. Predictions for Source Leaning seems to perform better than those for Bias Intensity and Expertise.

**Discussion**

The results of the Supplementary Study suggest the model’s predictions for source characteristics are effective, as they always correlate significantly with the observed posteriors, but are not as accurate as belief updating for hypotheses (from Study 1). While the VM scores for Source Leaning were effectively equal in size to the scores we observed for Study 1, performance was worse everywhere else. The Brier scores are higher, and the correlations lower. Predictions appear to be the worst for Expertise, and the best for Source Leaning – though the performance of the Bias Intensity and Expertise predictions also depended on the choice of how to set people’s priors, particularly for the correlations.

One problem noted in the visual inspection was that when the source’s testimony was that the policy had been beneficial, the predicted trend for Source Leaning appears to go in the *opposite* direction to what was observed. But given there is no equivalent error in the ‘Harmful’ testimony condition, and the observed linear trend of the evidence condition on posteriors was actually null, with an OLS regression coefficient *b* = 0.01 (*se* = 0.01), *t*(208) = .968, *p* = .334, we assume this is a random error rather than the kind of systematic discrepancy that would necessitate adjusting the model.

However, one further limitation of this study is that, as Figure S7 shows, there is some evidence that the correlation between observed and predicted posteriors was weaker for later trials for Bias Intensity and Source Leaning, indicating participants may have become inattentive later on, or that the repeated-measures design introduce carryover effects. A study which was able to avoid whatever it is that causes this effect might therefore find stronger correlations.


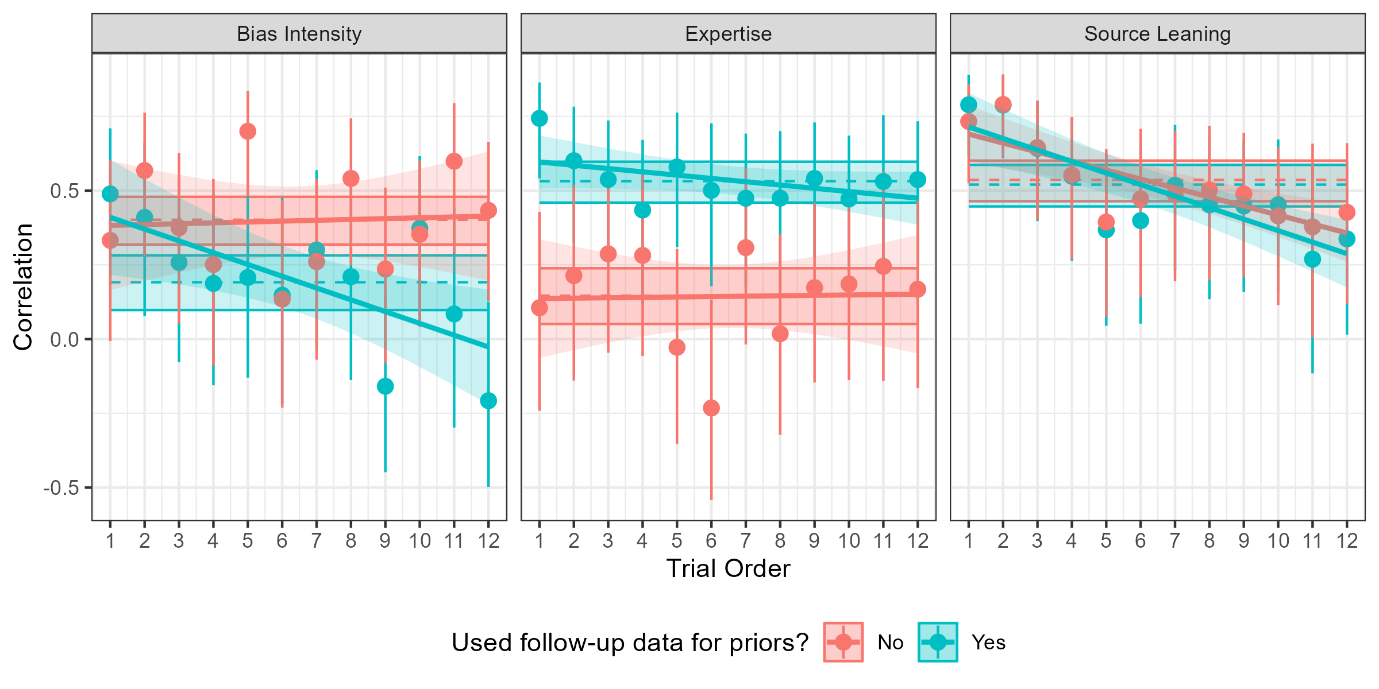


**Figure S7**

*The correlation between observed and predicted posteriors at different points within the trial.*

**Supplementary Material 4: Latin Square for Study 1 Randomisation**

Table 7 is the Latin Square used to assign policy areas to trials in Study 1 pseudo-randomly. Participants were randomised between Streams A and B and Branches 1-6. The policy for a given condition for a given branch was whichever policy is on the same row as that condition, in the column corresponding to the branch. High.E and Low.E refer to High and Low Expertise, respectively; Anti.L, Pro.L, and Imp.L refer to an Anti-Government, Pro-Government, and Neutral Source Leaning respectively; Anti.T and Pro.T refer to Anti-Government (“harmful”) and Pro-Government (“beneficial”) Testimony respectively; For. Trade is short for “Foreign Trade”. Very similar Latin Squares were used to assign stimuli to trials in the Supplementary Study.

**Table S7**

*Latin Square for assigning policy areas to trials, Study 1*.

| **Trial Condition** | | **Policy Area** | | | | | |
| --- | --- | --- | --- | --- | --- | --- | --- |
| **Stream A** | **Stream B** | **Branch 1** | **Branch 2** | **Branch 3** | **Branch 4** | **Branch 5** | **Branch 6** |
| High.E_Anti.L_Pro.T | High.E_Anti.L_Anti.T | Agricultural | Energy | For. Trade | Transport | Healthcare | Housing |
| High.E_Pro.L_Anti.T | High.E_Pro.L_Pro.T | Housing | Agricultural | Energy | For. Trade | Transport | Healthcare |
| High.E_Imp.L_Pro.T | High.E_Imp.L_Anti.T | Healthcare | Housing | Agricultural | Energy | For. Trade | Transport |
| Low.E_Anti.L_Anti.T | Low.E_Anti.L_Pro.T | Transport | Healthcare | Housing | Agricultural | Energy | For. Trade |
| Low.E_Pro.L_Pro.T | Low.E_Pro.L_Anti.T | For. Trade | Transport | Healthcare | Housing | Agricultural | Energy |
| Low.E_Imp.L_Anti.T | Low.E_Imp.L_Pro.T | Energy | For. Trade | Transport | Healthcare | Housing | Agricultural |

1. We again calculated correlation coefficients for each individual for their observed and predicted posteriors, then found the mean. These were from left to right: 0.45, 0.46, 0.57, 0.62, 0.26, 0.24. [↑](#footnote-ref-1)
